# Identification of PKP 2/3 as potential biomarkers of ovarian cancer based on bioinformatics and experiments

**DOI:** 10.1186/s12935-020-01602-3

**Published:** 2020-10-17

**Authors:** Lingling Gao, Xiao Li, Qian Guo, Xin Nie, Yingying Hao, Qing Liu, Juanjuan Liu, Liancheng Zhu, Limei Yan, Bei Lin

**Affiliations:** 1 Department of Obstetrics and Gynaecology, Shengjing Hospital of China Medical University, No.36 Sanhao Street, Heping District, Shenyang, 110004 Liaoning China; 2 Key Laboratory of Maternal-Fetal Medicine of Liaoning Province, Key Laboratory of Obstetrics and Gynecology of Higher Education of Liaoning Province, Shenyang, Liaoning China

**Keywords:** Ovarian cancer, PKP1/2/3, Prognosis, Biomarkers, Immune infiltration, Bioinformatics analysis

## Abstract

**Background:**

Plakophilins (PKPs) are widely involved in gene transcription, translation, and signal transduction, playing a crucial role in tumorigenesis and progression. However, the function and potential mechanism of PKP1/2/3 in ovarian cancer (OC) remains unclear. It’s of great value to explore the expression and prognostic values of PKP1/2/3 and their potential mechanisms, immune infiltration in OC.

**Methods:**

The expression levels, prognostic values and genetic variations of PKP1/2/3 in OC were explored by various bioinformatics tools and databases, and PKP2/3 were selected for further analyzing their regulation network and immune infiltration. Gene Ontology (GO) and Kyoto Encyclopedia of Genes and Genomes pathways (KEGG) enrichment were also conducted. Finally, the expression and prognosis of PKP2 were validated by immunohistochemistry.

**Results:**

The expression level and prognosis of PKP1 showed little significance in ovarian cancer, and the expression of PKP2/3 mRNA and protein were upregulated in OC, showing significant correlations with poor prognosis of OC. Functional enrichment analysis showed that PKP2/3 and their correlated genes were significantly enriched in adaptive immune response, cytokine receptor activity, organization of cell–cell junction and extracellular matrix; KEGG analysis showed that PKP2/3 and their significantly correlated genes were involved in signaling pathways including cytokine-mediated signaling pathway, receptor signaling pathway and pathways in cancer. Moreover, PKP2/3 were correlated with lymphocytes and immunomodulators. We confirmed that high expression of PKP2 was significantly associated with advanced stage, poor differentiation and poor prognosis of OC patients.

**Conclusion:**

Members of plakophilins family showed various degrees of abnormal expressions and prognostic values in ovarian cancer. PKP2/3 played crucial roles in tumorigenesis, aggressiveness, malignant biological behavior and immune infiltration of OC, and can be regarded as potential biomarker for early diagnosis and prognosis evaluation in OC.

## Background

Ovarian cancer (OC) is one of the most common gynecological malignancy in female reproductive system worldwide, which has the characteristics of high metastasis, chemotherapy resistance and recurrence after surgery. Over 70% of the patients are in the advanced stage at age of diagnosed [1, 2]. In recent years, the incidence and mortality rates of patients with OC have significantly increased. Although the combination of chemotherapy and radiotherapy has been widely promoted in clinical practice, the 5 years survival rate of OC patients is still less than 40% [3]. Therefore, a better understanding of potential molecular mechanisms provides opportunities in the early diagnosis and prognosis assessment of OC.

Plakophilins (PKPs) are members of subfamily p120ctn (p120-catenins) of the armadillo protein family, which mainly includes p120ctn (p120ctn, CTNND1), NPRAP/δ-catenin (CTNND2), ARVCF (armadillo repeat gene deleted in velocardio-facial syndrome), p0071/ plakophilin (PKP4), and plakophilin 1/2/3 (PKP1/2/3), while all members of the p120ctn family have characteristic armadillo repeat sequences (arm-repeats), which are flanked by N-terminal head domain and C-terminal tail domain. These molecules play a crucial role in regulating protein binding, cell junction (desmosome and adhesion junction) and signal transduction [4]. PKP1/2/3 were mainly located in desmosomes, and regulated the stability of desmosomes, cell junctions and adhesion [5, 6]. Researchers found that PKP1/2/3 participated in regulating cell migration and affected the development of tissue and embryo. In mice, knockout of *PKP2* caused unstable connections among myocardial cells, resulting in myocardial trabecularization and hemorrhage, circulatory system failure and finally led to death [7], and knockout of *PKP3* generated abnormal development and morphology of hair follicles and histological inflammation [8]. In humans, mutation of *PKP1* gene caused a skin fragility syndrome characterized by ectodermal dysplasia and skin fragility [9, 10], mutation of *PKP2* gene was closely associated with arrhythmogenic right ventricular cardiomyopathy(ARVC) [11].

In recent years, many studies have shown that PKP1/2/3 played a well-established role in the occurrence, development, invasion and metastasis of various malignancy, including breast cancer, prostate cancer, gastric cancer [12]. PKP3 could participate in the invasion and metastasis of ovarian cancer [13, 14]. However, the expression, prognostic value, biological function and molecular mechanism of PKP1/2/3 in ovarian cancer are not clearly clarified. In this study, on the basis of various bioinformatics databases, we comprehensively and objectively analyzed the relationship between PKP1/2/3 and the occurrence, development, prognostic value and immune infiltration of OC, as well as the related functional regulatory network. As the expression and regulatory mechanism of PKP3 in ovarian cancer has been confirmed by many researchers previously [13, 14], the expression and prognosis of PKP2 were further selected for validation with immunohistochemistry, which is helpful to provide a new strategy for early diagnosis and treatment of ovarian cancer.

## Methods

### Oncomine database

Oncomine database (https://www.oncomine.org) [15] is the largest oncogene chip database worldwide, including 715 gene datasets and 86,733 samples. We used Oncomine database to analyze the expression of PKP1/2/3 mRNA in cancer tissues and normal tissues. This study was carried out according to the criteria as follows: ① Cancer Type: ovarian cancer; ② Gene: PKP1/2/3; ③ Analysis Type: Cancer vs. Normal Analysis; ④ Data Type: mRNA; ⑤ thresholds: *P* value < 0.01, Fold change > 2 and gene rank = top 10%.

### GEPIA database

GEPIA2 (Gene Expression Profiling Interactive Analysis) (https://gepia2.cancer-pku.cn/) (Version 2.0) [16] is a highly visual analysis website that contains RNA sequencing expression data of 9736 tumor samples and 8587 normal samples from TCGA and GTEx. The database includes a variety of analysis modules such as differential gene expression of tumor tissue and adjacent normal tissue, survival and prognosis analysis, as well as correlation and dimensionality reduction analysis. In this study, GEPIA database was used to analyze the expression of PKP1/2/3 in ovarian serous cystadenocarcinoma. The filter criteria were as follows: |Log2FC| Cutoff: 2; *P* value Cutoff: 0.05.

### Human Protein Atlas (HPA)

Human Protein Atlas (https://www.proteinatlas.org/) (updated date: 2020-03-06) [17] is a free public platform which provides the distribution information of 24,000 human proteins in tissues and cells. The database can provide the location, expression and prognosis of proteins in 48 normal tissues, 20 tumor tissues, 47 cell lines and 12 blood cells validated by professionals with immunology method. In this study, HPA database was used to analyze the immunohistochemical staining of PKP1/2/3 in ovarian cancer tissues.

### Kaplan–Meier Plotter

Kaplan–Meier Plotter (https://kmplot.com) (updated date: 2020-05-25) [18] is an online tool that can be used to evaluate the prognosis of 54,675 genes in 10,188 cancer samples, including 4142 cases of breast cancer, 1648 cases of ovarian cancer, 2437 cases of lung cancer and 1065 cases of gastric cancer. In this study, we explored the prognostic values of PKP1/2/3 mRNA in ovarian cancer by Kaplan–Meier Plotter. The prognostic values of high and low PKP1/2/3 expression were evaluated by hazard ratio (HR) with 95% confidence interval (CIs), and log rank *P*-value. The filter criteria were as follows: ① Cancer Type: ovarian cancer; ② Gene: PKP1/2/3; ③ Survival: OS/PFS; ④ Follow up threshold: 120 months; ⑤ Split patients by: the auto selected best cut-off.

### CBioPortal

CBioPortal for Cancer Genomics (https://www.cbioportal.org) [19] is a public available resource for interactive exploration of multiple cancer genomics database derived from TCGA, ICGC and GEO. The integrated genomic data include somatic mutation, DNA copy number change (CNAs), mRNA expression, DNA methylation and protein abundance. We analyzed genetic alterations of PKP1/2/3 in Ovarian Serous Cystadenocarcinoma (TCGA, Provisional) with cBioPortal. The genomic profiles included mutations, putative copy-number alterations (CNA) from GISTIC, mRNA expression z-scores (RNA Seq V2 RSEM) and protein expression Z-scores (RPPA). Neighboring genes and network was calculated according to the cBioPortal's online instruction.

### GeneMANIA analysis

GeneMANIA (https://www.genemania.org) [20] shows a flexible accessible web interface that facilitates prediction and interaction of genes. GeneMANIA could construct protein–protein interaction (PPI) network, protein-DNA interaction, signal pathway, physiological and biochemical response, and the data are updated regularly. GeneMANIA was used to construct and visualize the functions and PPI network of PKP1/2/3 and their related molecules.

### LinkedOmics analysis

LinkedOmics (https://www.linkedomics.org/login.php) (updated date: 2018-10-08) [21] provides multi-dimensional datasets and clinical datasets based on a web platform, containing sample data from 32 cancer types and a total of 11,158 patients in the TCGA database. The LinkFinder module of LinkedOmics is performed to explore functions, pathway and interaction network of the differentially expressed genes associated with PKP1/2/3 in TCGA_OV dataset (n = 303). All the results were presented in the form of volcano map, heat map and scatter map. The filter criterions were as follows: cancer cohort: TCGA_OV; dataset: TCGA_OV (RNAseq); dataset attribute: PKP1/2/3; search target dataset: TCGA_OV (RNAseq); statistical method: Pearson correlation test. The false discovery rate (FDR) < 0.05, and 500 simulations were carried out.

### Metascape

Metascape (https://metascape.org) (Update Date: 2020-03-20) [22] is an online tool for gene annotation and analysis, which integrates GO, KEGG, UniProt, Drugbank and other authoritative database. The tool performs not only pathway enrichment and biological process annotation, but also protein interaction network analysis. In this study, Metascape was employed to complete Gene Ontology (GO) and Kyoto Encyclopedia of Genes and Genomes pathways (KEGG) enrichment of PKP2/3 and their related differentially expressed genes. Restrictions: *P* < 0.01, a minimum count of 3, enrichment factor > 1.5 were considered to be statistically significant.

### TISIDB analysis

TISIDB (https://cis.hku.hk/TISIDB) [23] database integrates 988 immune-associated anti-tumor genes, high-throughput screening techniques, molecular profiles, and paracancerous multi-omics data, as well as various immunological data developed from 7 public databases. The database facilitates analysis of correlations between selected genes and lymphocytes, immune regulators and chemokines. In this study, TISIDB database was used to analyze the relationships between levels of PKP2/3 expression and lymphocyte, immunomodulators.

### Patients and paraffin-embedded tissue samples

The study was approved by the Ethics Committee of China Medical University. A total of 170 patients and paraffin-embedded specimens were collected after operation from Department of Obstetrics and Gynecology of Shengjing Hospital of China Medical University from 2004 to 2018. The pathological diagnosis of all tissue sections were confirmed by in-house experts, as follows: malignant group, n = 117; borderline group, n = 23; benign group, n = 15; normal group, n = 15 (Table 2). The average ages of patients in the malignant group, borderline group, benign group, and normal group were 54 years old (19–83), 44 years old (22–84), 44 years old (13–79), and 45 years old (36–57), respectively. There was no significant difference among the average ages of each group (*P* > 0.05). The pathological types of ovarian cancer were as follows: serous adenocarcinoma (n = 76), mucinous adenocarcinoma (n = 13), endometrioid carcinoma (n = 20), clear cell carcinoma (n = 8). In malignant group, there were 35, 29, 53 cases of well, moderate, and poor differentiation, respectively. According to the criteria of the International Federation of Gynecology and Obstetrics (FIGO, 2009), the pathological stages were judged as follows: FIGO stage I-II (50 cases) and FIGO stage III-IV (67 cases). Lymphnode metastasis was judged as follows: no metastasis (70 cases), metastasis (24 cases), and no lymphadenectomy (23 cases). All of the patients had primary ovarian cancer with complete clinical and pathologic data, and patients with chemotherapy, radiotherapy, and hormone therapy before surgery were not implemented in this study.

### Immunohistochemistry

The ovarian tissue specimens were fixed with 4% formalin and embedded in paraffin, then dissected using 5-μm serial consecutive sections [24]. The expression of PKP2 in ovarian tissues was detected by immunohistochemical streptavidin-peroxidase (SP) staining (Ultrasensitive™ SP (Mouse/Rabbit) IHC Kit, Maixin, China) and the working concentration of primary antibody against PKP2 was 1:100 (Affinity, China, Cat# DF7385). Human heart muscle tissue served as positive control, phosphate-buffered saline (PBS) served as a negative control instead of primary antibody. The empirical procedure was performed based on the manufacturer's instructions. The presence of buffy granules in the cell were considered as positive. According to the chromatosis intensity, no pigmentation, light yellow, buffy, and brown are scored 0, 1, 2, and 3, respectively. The percentage of pigmented cells in the visual field was as follows: less than 5% are 0, 5–25%: 1, 26–50%: 2, 51–75%: 3, and greater than 75%: 4. The final score was obtained by multiplying the scores of the chromatosis intensity and the percentage of stained cells obtained the following scores: 0–2 scores ( −); 3–4 scores ( +); 5–8 scores (+ +); and 9–12 scores (+ + +). Two pathologists examined the sections independently to control error.

### Statistical analysis

All data were statistically analyzed using the SPSS 21.0 software (IBM Corporation, Armonk, NY, USA) and expressed as the mean ± standard deviation. The data were analyzed with *t*-test and Chi-squared test. The survival analysis was conducted using the Kaplan–Meier and log-rank test. The Cox model was adopted to analyze the prognosis of patients. Bilateral *P* values < 0.05 was considered statistically significant difference.

## Results

### Levels of PKP1/2/3 mRNA in various cancers analyzed by Oncomine database

**Fig. 1:**
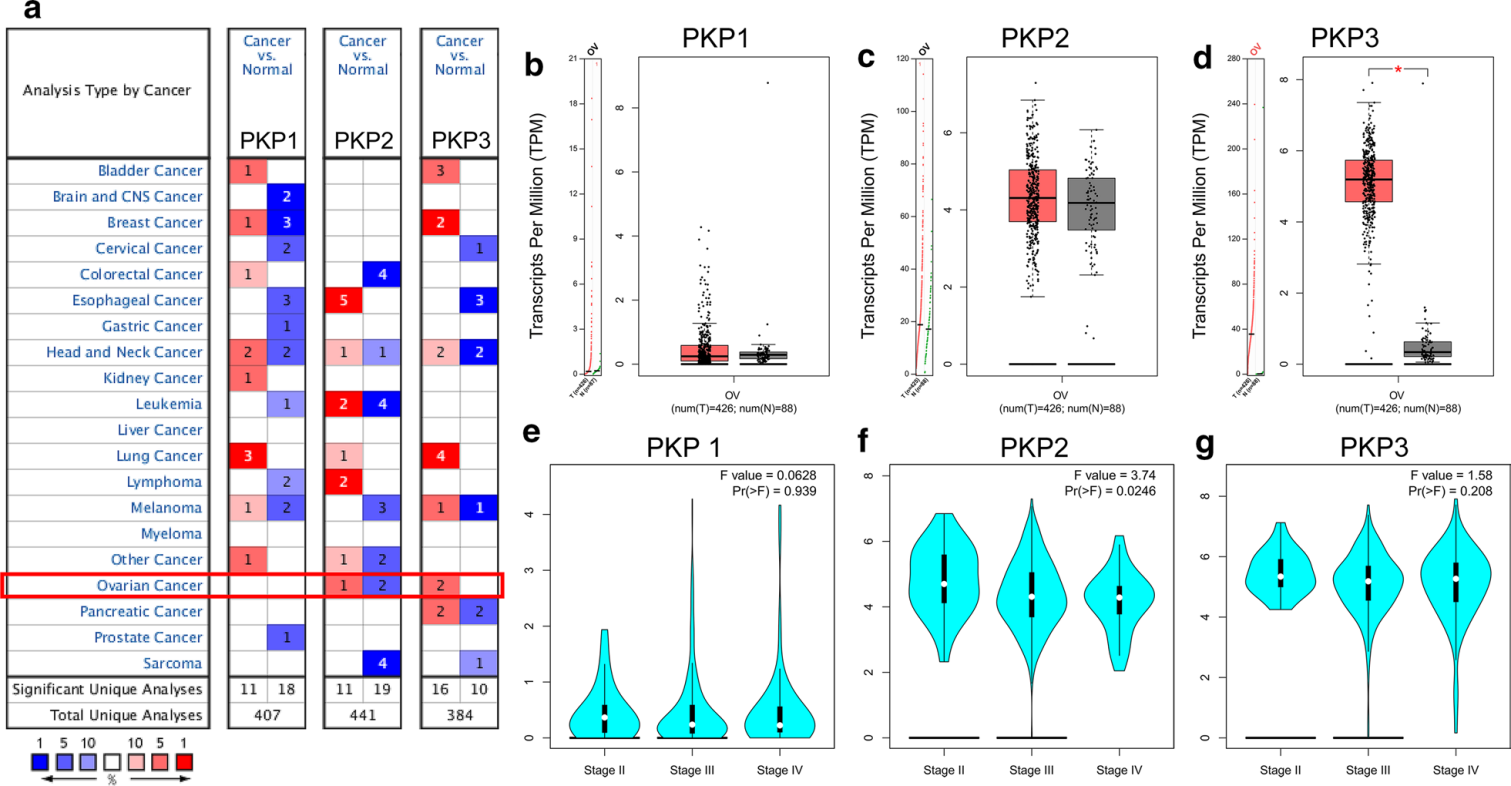
The expression of PKP1/2/3 mRNA in OV and correlation between PKP1/2/3 expression and tumor stages in OV (Oncomine and GEPIA). **a** The expression of PKP1/2/3 in ovarian cancer explored by Oncomine database. **b–d** The mRNA expression of PKP1 (**b**), PKP2 (**c**) and PKP3 (**d**) in OV and normal tissues from GEPIA dataset. **e–g** The mRNA expression of PKP1 (**e**), PKP2 (**f**) and PKP3 (**g**) in different tumor stages in patients with OV in GEPIA dataset. *P < 0.05, indicating that the results are statistically significant. **P < 0.01, ***P < 0.001. *PKP* plakophilin, *OV* Ovarian serous cystadenocarcinoma, *GEPIA* Gene Expression Profiling Interactive Analysis

To determine the role of PKP1/2/3 in tumorigenesis and development in ovarian cancer, we detected the levels of PKP1/2/3 mRNA in ovarian cancer tissues and normal tissue by Oncomine database. According to the screening criteria, there was no significant difference in the level of PKP1 mRNA between ovarian cancer and normal tissues. In Yoshihara’s dataset, the level of PKP2 mRNA were significantly upregulated in malignant group compared with normal tissues (Fold change = 7.903, *P* = 7.48E−07). The transcription level of PKP3 were significantly higher in ovarian serous adenocarcinoma than those in the normal tissues in Yoshihara’s dataset (Fold change = 62.784, *P* = 2.34E−09). In Bonome’s dataset, the level of PKP3 mRNA was also upregulated in ovarian carcinoma compared with normal group (Fold change = 2.459, *P* = 4.35E−09) (Fig. 1a, Table 1).

**Table 1:** The expression of PKP1/2/3 mRNA in different types of ovarian cancer and normal tissues (Oncomine)

Types of ovarian cancer (cases) vs. normal tissues (cases)	Fold change	*t*-test	*P* value	Ref
PKP1				
Ovarian serous cystadenocarcinoma (586) vs. Ovary (8)	1.087	4.984	4.49E−04	TCGA ovarian
Ovarian clear cell adenocarcinoma (8) vs. Ovary (4)	1.152	3.743	2.00E−03	Hendrix ovarian
Ovarian mucinous adenocarcinoma (13) vs. Ovary (4)	1.084	3.01	1.10E−02	
Ovarian serous adenocarcinoma (41) vs. Ovary (4)	1.054	2.231	4.30E−02	
Ovarian serous surface papillary carcinoma (28) vs. Ovary (4)	1.178	1.755	4.50E−02	Welsh ovarian
Ovarian endometrioid adenocarcinoma (9) vs. Ovarian surface epithelium (5)	1.184	2.182	0.025	Lu ovarian
Ovarian carcinoma (185) vs. Ovarian surface epithelium (10)	1.28	5.551	7.19E−05	Bonome ovarian
PKP2				
Ovarian serous adenocarcinoma (43) vs. Peritoneum (10)	7.903	8.409	7.48E−07	Yoshihara Ovarian
Ovarian carcinoma (185) vs. Ovarian surface epithelium (10)	1.157	4.37	4.37E−04	Bonome ovarian
Ovarian mucinous adenocarcinoma (13) vs. Ovary (4)	1.195	2.126	4.00E−02	Hendrix ovarian
PKP3				
Ovarian serous cystadenocarcinoma (586) vs. Ovary (8)	1.28	2.071	3.70E−02	TCGA ovarian
Ovarian serous adenocarcinoma (43) vs. Peritoneum (10)	62.784	14.553	2.34E−09	Yoshihara ovarian
Ovarian carcinoma (185) vs. Ovarian surface epithelium (10)	2.459	11.985	4.35E−09	Bonome ovarian
Ovarian serous adenocarcinoma (6) vs. Ovary (4)	1.871	5.866	6.07E−04	Adib ovarian
Ovarian mucinous adenocarcinoma (13) vs. Ovary (4)	1.489	9.539	6.64E−07	Hendrix ovarian
Ovarian clear cell adenocarcinoma (8) vs. Ovary (4)	1.338	6.555	3.92E−05	
Ovarian serous adenocarcinoma (41) vs. Ovary (4)	1.352	8.728	2.92E−05	
Ovarian endometrioid adenocarcinoma (37) vs. Ovary (4)	1.318	8.488	1.20E−04	
Ovarian clear cell adenocarcinoma (7) vs. Ovarian surface epithelium (5)	1.65	3.636	5.00E−03	Lu ovarian
Ovarian mucinous adenocarcinoma (9) vs. Ovarian surface epithelium (5)	1.458	2.509	1.80E−02	
Ovarian serous adenocarcinoma (20) vs. Ovarian surface epithelium (5)	1.6	3.574	7.00E−03	
Ovarian endometrioid adenocarcinoma (9) vs. Ovarian surface epithelium (5)	1.455	2.661	1.70E−02	

### Differential expression levels of PKP1/2/3 mRNA and protein in OC detected by GEPIA and Human Protein Atlas

**Fig. 2:**
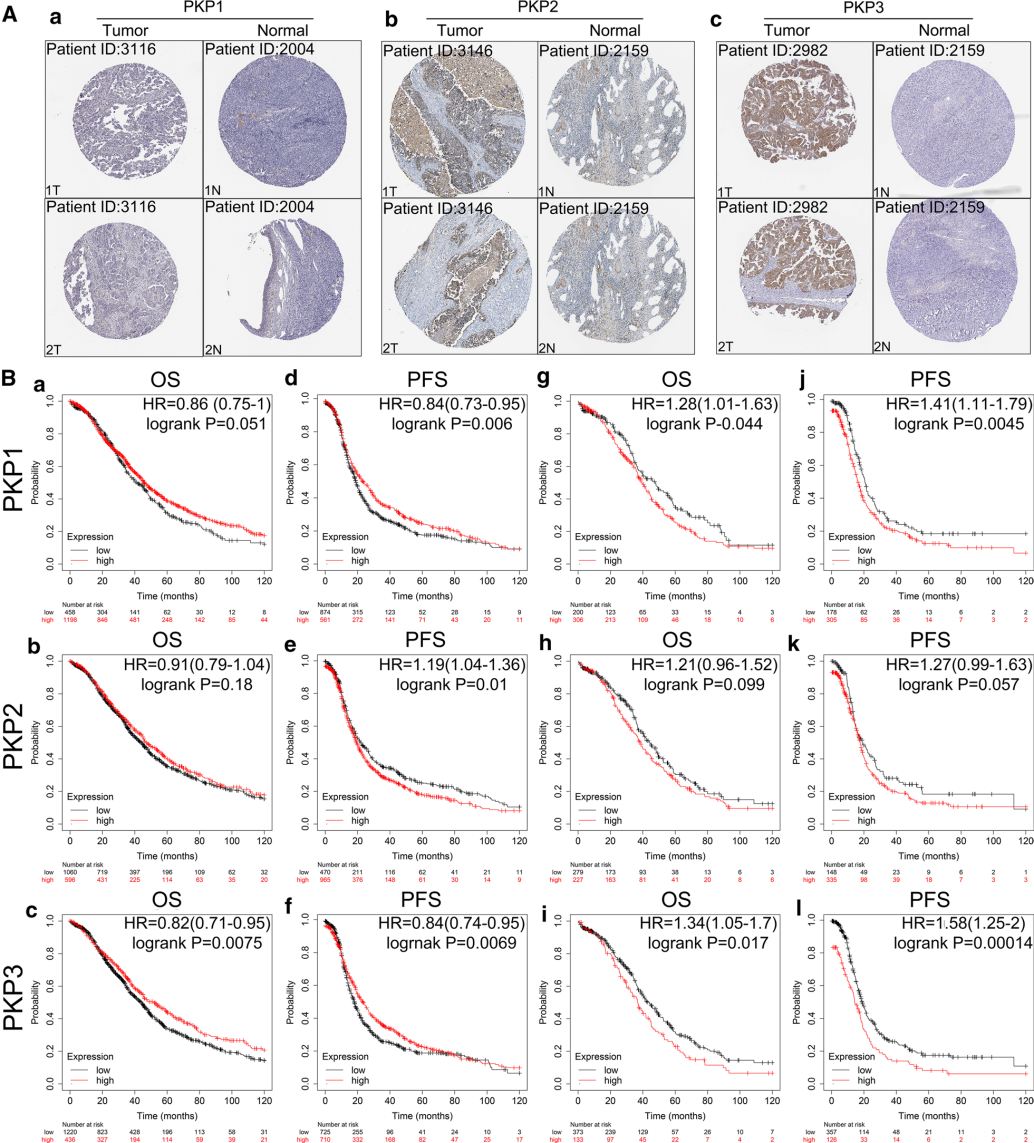
The expression and prognostic values of PKP1/2/3 protein in ovarian cancer (Human Protein Atlas and Kaplan–Meier plotter).** A** The expression of PKP1 (**a**), PKP2 (**b**) and PKP3 (**c**) in OC and normal tissues stained by immunohistochemistry. **B** (**a–c**) Relationship between the expression of PKP1 (**a**), PKP2(**b**), PKP3(**c**) and OS in patients with OC; (**d**–**f**) Relationship between the expression of PKP1 (**d**), PKP2 (**e**), PKP3( **f**) and PFS in patients with OC; (**g**–**i**) Relationship between the expression of PKP1 (**g**), PKP2 (**h**), PKP3 (**i**) and OS in OC patients with *TP53* mutation; (**j**–**l**) Relationship between the expression of PKP1 (**j**), PKP2 (**k**), PKP3 (**l**) and PFS in OC patients with *TP53* mutation. *PKP* plakophilin, *OC* ovarian cancer, *OS* Overall Survival, *PFS* progression-free survival

Ovarian serous cystadenocarcinoma is the most common pathological type of ovarian cancer [25]. We further investigated the expression of PKP1/2/3 in Ovarian serous cystadenocarcinoma (OV) and normal ovarian tissue with the GEPIA database. The results showed that the expression level of PKP3 was significantly higher in OV than that in normal tissues (*P* < 0.05), while the expression levels of PKP1 and PKP2 were not statistically significant between OV and normal tissues (*P* > 0.05) (Fig. 1b–d). The expression levels of PKP2 in Yoshihara's dataset and GEPIA database were not fully consistent, the main reasons may be as follows: the different samples size, ages, regions and control groups. We further explored the relationship between PKP1/2/3 mRNA and tumor stages in OV. The results suggested that the level of PKP2 mRNA was associated with the tumor stages (*P* < 0.05), while the levels of PKP1 and PKP3 mRNA were not significantly different among the tumor stages (*P* > 0.05) (Fig. 1e–g). We further verified the expression of PKP1/2/3 protein in ovarian cancer tissues and normal tissues by immunohistochemistry with The Human Protein Atlas database. The results showed that PKP1 protein was not detected in ovarian cancer tissues and normal tissues; the levels of PKP2 and PKP3 proteins were significantly higher in ovarian cancer tissues than those in normal tissues, PKP2 protein was mainly located in nuclear, cell membrane and cytoplasm, and PKP3 protein was mainly located to the cell membrane and cytoplasm (Fig. 2A (a–c), Additional file 1: Table S1).

### Prognostic values of PKP1/2/3 in patients with OC by Kaplan–Meier Plotter

The prognostic values of PKP1/2/3 in patients with OC were explored by Kaplan–Meier Plotter, and the truncated data were set to overall survival (OS) and progression-free survival (PFS), a total of 1657 and 1436 cases in the database met the standards, respectively. The results revealed that the levels of PKP1/2 mRNA had no significant correlation with OS in patients with OC (all *P* > 0.05) (Fig. 2B (a, b)), and low expression of PKP1 mRNA showed significant correlations with poor PFS in patients with OC [HR = 0.84 (0.73–0.95), log-rank *P* = 0.006] (Fig. 2B (d)). High expression of PKP2 showed significant correlations with poor PFS in patients with OC [HR = 1.19 (1.04–1.36), log-rank *P* = 0.01] (Fig. 2B (e)), while high expression of PKP3 mRNA displayed better OS [HR = 0.82 (0.71–0.95), log-rank *P* = 0.0075] and PFS [HR = 0.84 (0.74–0.95), log-rank *P* = 0.0069] in patients with OC (Fig. 2B (c) and Fig. 2B (f)). We further detected the correlations of PKP1/2/3 with OS and PFS in ovarian cancer patients with *TP53* mutation, the results showed that high expression of PKP1 had poor OS [HR = 1.28 (1.01–1.63), log-rank *P* = 0.044] and PFS [HR = 1.41(1.11–1.79), log-rank *P* = 0.0045] in *TP53* mutated ovarian cancer patients (Fig. 2B (g) and Fig. 2B (j)) (Additional file 1: Table S2). In addition, high expression of PKP3 had poor OS [HR = 1.34 (1.05–1.7), log-rank *P* = 0.017] and PFS [HR = 1.58(1.25–1.2), log-rank *P* = 0.00014] in *TP53* mutated ovarian cancer patients (Fig. 2B (i) and Fig. 2B (l)).

### Genomic alteration and co-expression gene network of PKP1/2/3 in ovarian cancer with cBioPortal and GeneMANIA

**Fig. 3:**
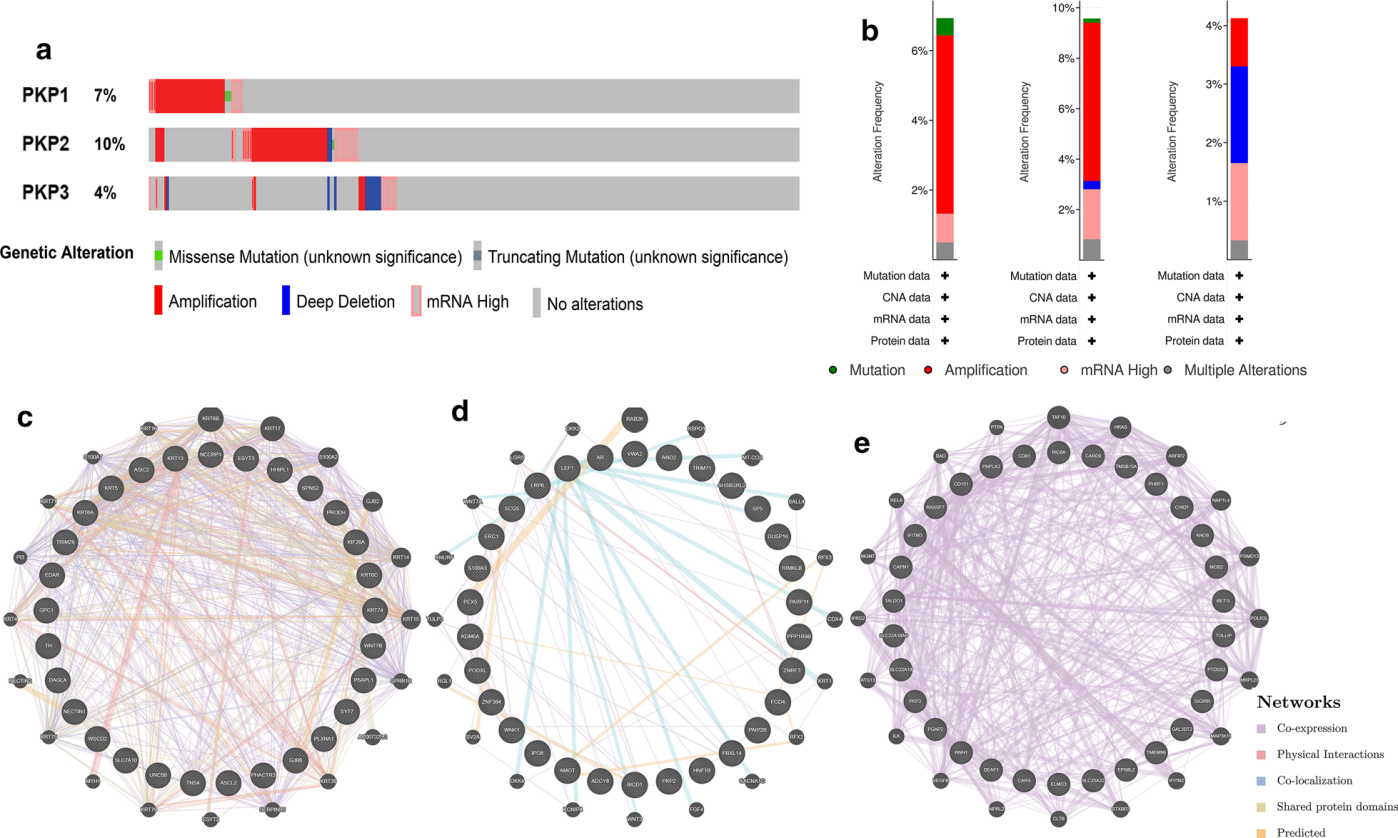
Mutation and interaction analysis of PKP1/2/3 in OC (cBioPortal and GeneMANIA). **a**, **b** Genes expression and mutation analysis of PKP1/2/3 in OC. **c**–**e** Co-expression and interaction analysis of PKP1/2/3 in OC. *PKP* plakophilin, *OC* ovarian cancer

Many studies have shown that the frequency of genomic mutation was associated with the occurrence and development of tumors [26]. We analyzed the genetic variations, correlations, and network of PKP1/2/3 in ovarian cancer with cBioPortal based on the Ovarian Serous Cystadenocarcinoma (OV) (TCGA, Provisional) database, there were 144 samples (21%) with genetic alteration in PKP1/2/3, and the genetic alteration of PKP1/2/3 varied from 4 to 10% (PKP1, 7%; PKP2, 10%; PKP3, 4%) (Fig. 3a). PKP2 displayed the highest incidence of genetic variation (amplification, 6.27%; deep deletion, 0.33%; mutation, 0.17%; mRNA high, 1.98%; multiple alterations, 0.83%, respectively), both PKP1 and PKP3 indicated amplification events (Fig. 3b). The molecules closely correlated with the function of PKP1/2/3 were also detected by cBioPortal, and the top 50 co-expression genes were used to construct protein interaction network by GeneMANIA, which suggested that KRT13, NECTIN1 and HHIPL1 were closely related to the function of PKP1; ADCY8, SP5 and PPP1R9B were significantly associated with PKP2; PKP3 was associated with EPS8L2, ANO9 and RASSF7 (Fig. 3c–e).

### Interaction analyses of significant genes correlated with PKP2/3 in OC by LinkedOmics

**Fig. 4:**
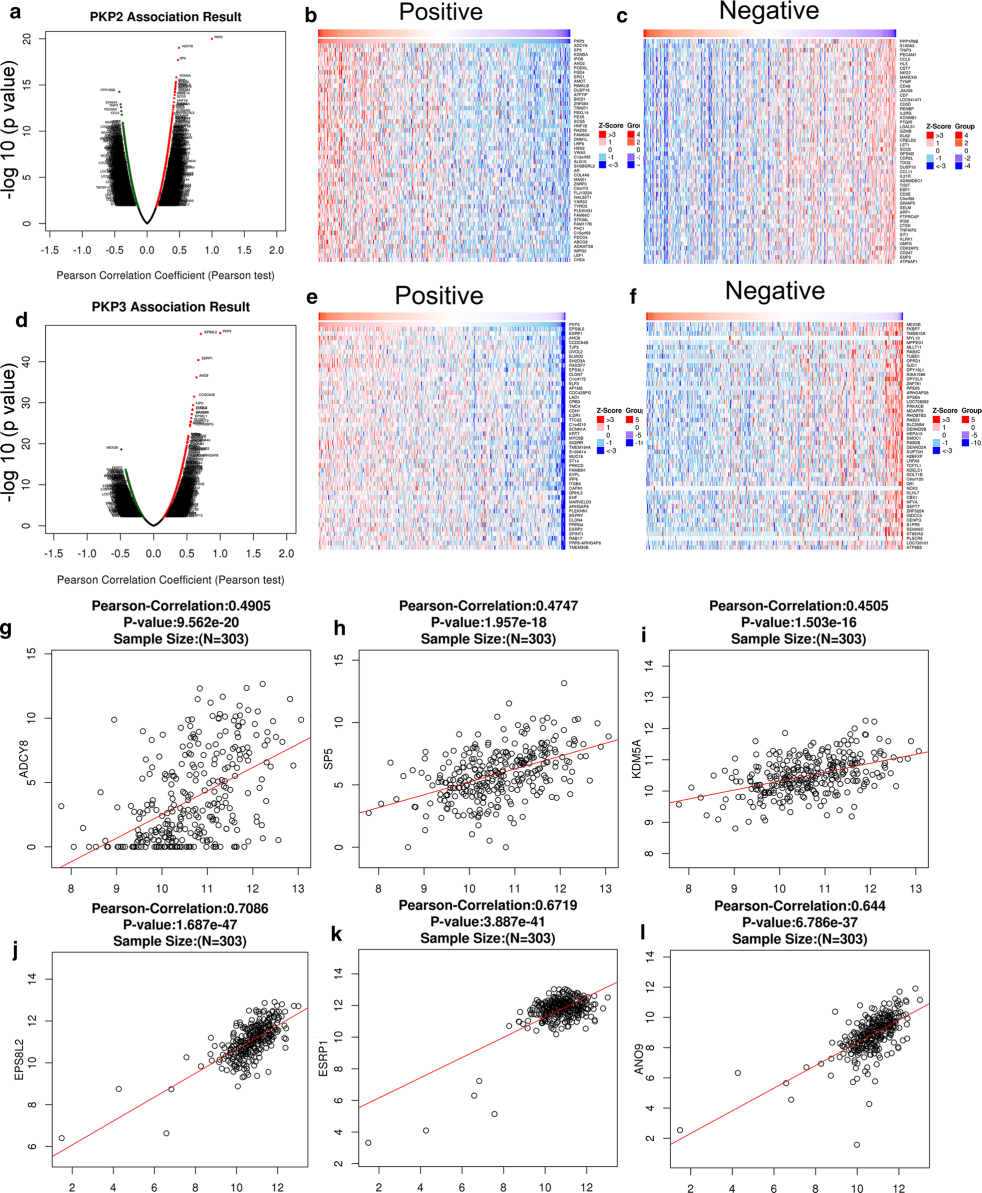
Differentially expressed genes in correlation with PKP2/3 in OV (LinkedOmics).** a–c** Positive and negative correlated gene of PKP2 in OV with volcano map and heat maps (TOP 50). **d–f** Positive and negative correlated gene of PKP3 in OV with volcano map and heat maps (TOP 50). **g–i** Significant genes (ADCY8, SP5 and KDM5A) correlated with PKP2 expression in the scatter plot. **j–l** Significant genes (EPS8L2, ESRP1 and ANO9) correlated with PKP3 expression in the scatter plot. Red indicates positive correlated genes and green indicates negative correlated genes. All y-axes represent “− log 10 (*p* value)”. *OV* Ovarian serous cystadenocarcinoma

The mRNA sequencing data of 303 patients with OV in TCGA database were analyzed by Function module in LinkedOmics. As shown in the volcano map, there were 2710 genes positively correlated with PKP2 (dark red dots) and 1850 genes negatively correlated with PKP2 (dark green spots) (FDR < 0.01) (Fig. 4a). The statistical scatter plot of individual gene indicated that the expression of PKP2 was positively correlated with ADCY8 (Pearson correlation = 0.4905, *P* = 9.562e−20), SP5 [Pearson correlation = 0.4747, *P* = 1.957e−18) and KDM5A (Pearson correlation = 0.4505, *P* = 1.503e−16)] (Fig. 4g–i). There were 1434 genes positively correlated with PKP3 (dark red dots) and 1875 genes negatively correlated with PKP3 (dark green spots) (FDR < 0.01) (Fig. 4d). The statistical scatter plot of individual gene suggested that the expression of PKP3 was positively correlated with EPS8L2 (Pearson correlation = 0.7086, *P* = 1.687e−47), ESRP1 (Pearson correlation = 0.6719, *P* = 3.887e−41) and ANO9 (Pearson correlation = 0.644, *P* = 6.786e−37) (Fig. 4j–l). Heat maps exhibited the top 50 gene sets which had significantly positive and negative correlation with PKP2/3, respectively (Fig. 4b–c and Fig. 4e–f). The results showed that PKP2/3 played an extensive role in regulating cell adhesion, protein activity and extracellular matrix organization.

### Functional and KEGG pathway enrichment analysis of PKP2/3 in OC

**Fig. 5:**
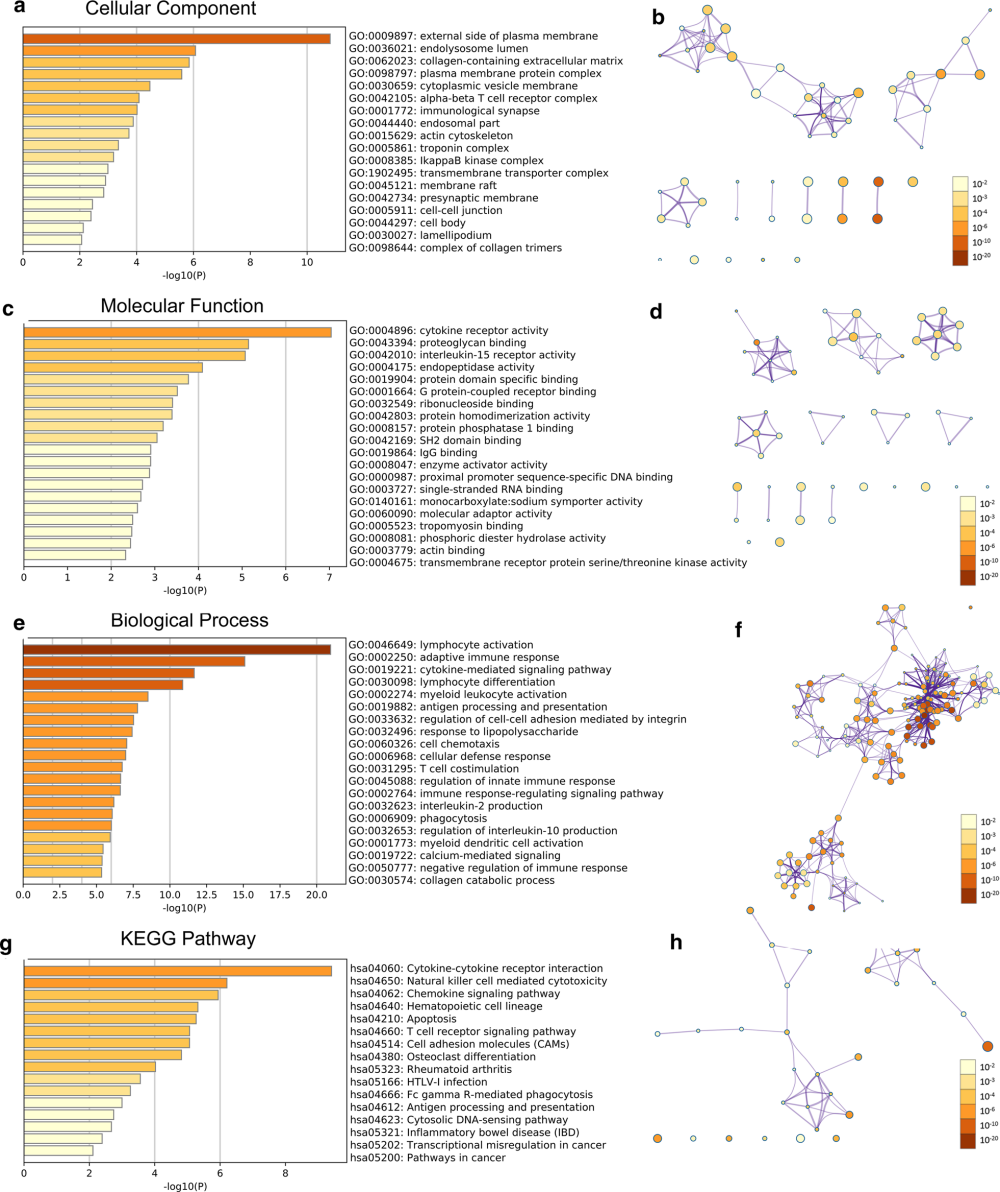
Significantly enriched GO function and KEGG pathways of PKP2 and co-expression genes in OV (Metascape). **a**, **b** Bar graph and network of cellular component enrichment colored by *P* value (Top 20). **c**, **d** Bar graph and network of molecule function enrichment colored by *P* value (Top 20). **e**, **f** Bar graph and network of biological process enrichment colored by *P* value (Top 20). **g**, **h** Bar graph and network of KEGG enriched terms colored by *P* value. *GO* Gene Ontology; *KEGG* Kyoto Encyclopedia of Genes and Genomes. Above results were colored by *P* value, where terms containing more genes tend to have a more significant *P* value

**Fig. 6:**
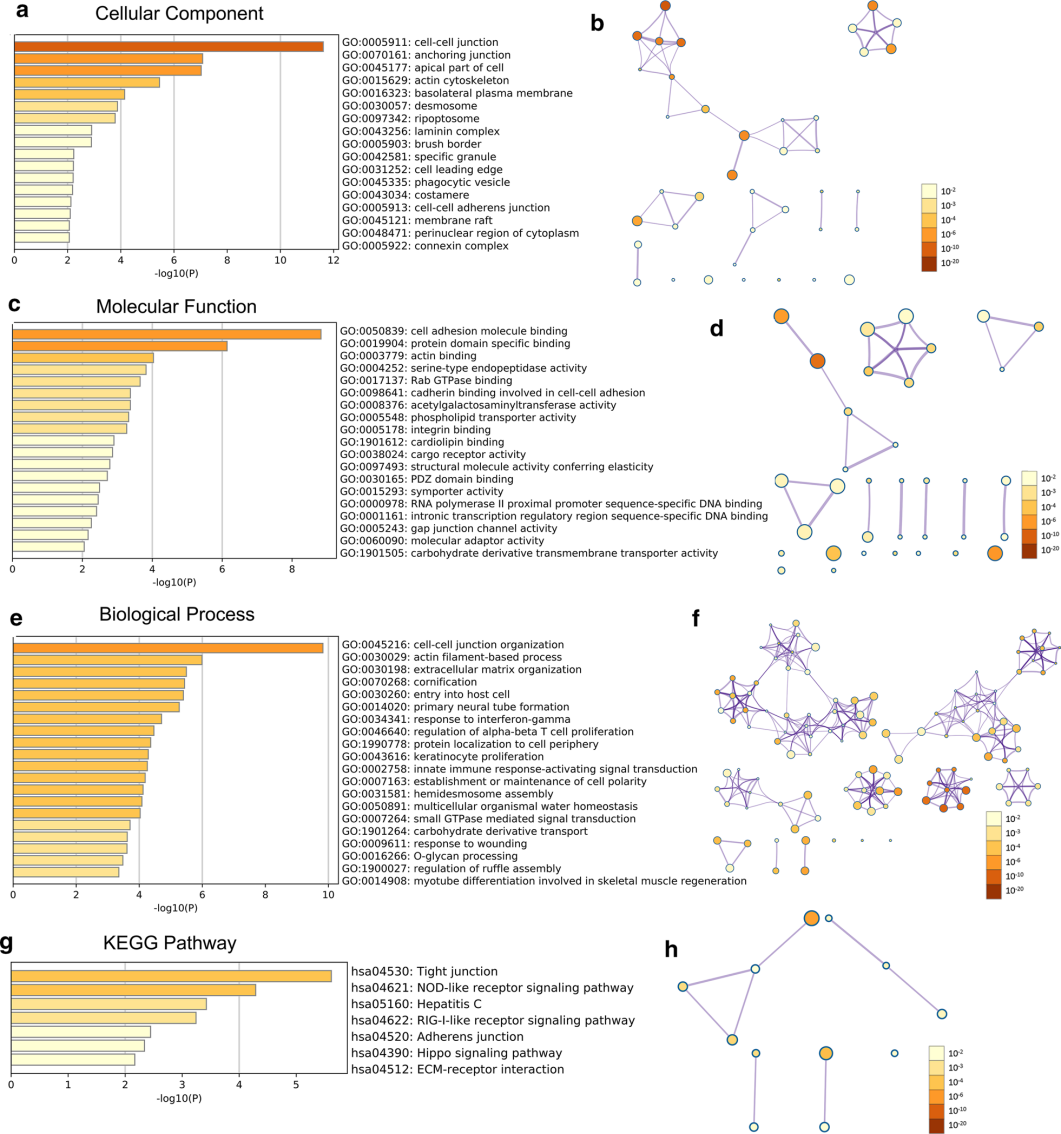
Significantly enriched GO function and KEGG pathways of PKP3 and co-expression genes in OV (Metascape). **a**, **b** Bar graph and network of cellular component enrichment colored by *P* value (Top 20). **c**, **d** Bar graph and network of molecule function enrichment colored by *P* value (Top 20). **e**, **f** Bar graph and network of biological process enrichment colored by *P* value (Top 20). **g**, **h** Bar graph and network of KEGG enriched terms colored by *P* value. *GO* Gene Ontology, *KEGG* Kyoto Encyclopedia of Genes and Genomes. Above results were colored by *P* value, where terms containing more genes tend to have a more significant *P* value

The potential function and mechanism of PKP2/3 and their significantly correlated genes were analyzed by Metascape. GO displayed that PKP2 and its associated differentially expressed genes were mainly located in external side of plasma membrane, endo-lysosome lumen and collagen-containing extracellular matrix et al. (Fig. 5a, b and Additional file 1: Table S3). The molecular functions of PKP2 and their associated genes involved in regulating cytokine receptor activity, endopeptidase activity, proteoglycan binding and protein domain specific binding et al. (Fig. 5c, d and Additional file 1: Table S4). Above genes participated in biological processes such as adaptive immune response, cell–cell adhesion and lymphocyte differentiation et al. (Fig. 5e, f and Additional file 1: Table S5). PKP3 and its related differentially expressed genes were mainly located in cell–cell junction, anchoring junction and actin cytoskeleton (Fig. 6a, b and Additional file 1: Table S7). The molecular functions and biological processes of PKP3 and its associated genes involved in binding adhesion molecule, actin and protein domain, organizing cell–cell junction and extracellular matrix et al. (Fig. 6c–f and Additional file 1: Tables S8 and S9). The KEGG enrichment analysis showed that the signaling pathways in which PKP2 and its related differentially expressed genes participated included cytokine-cytokine receptor interaction, natural killer cell mediated cytotoxicity and chemokine signaling pathway et al. (Fig. 5g, h and Additional file 1: Table S6). PKP3 and its related differentially expressed genes involved in tight junction, NOD-like and RIG-I-like receptor signaling pathway (Fig. 6g, h and Additional file 1: Table S10).

### Correlation analysis between PKP2/3 expression and immune molecules

**Fig. 7:**
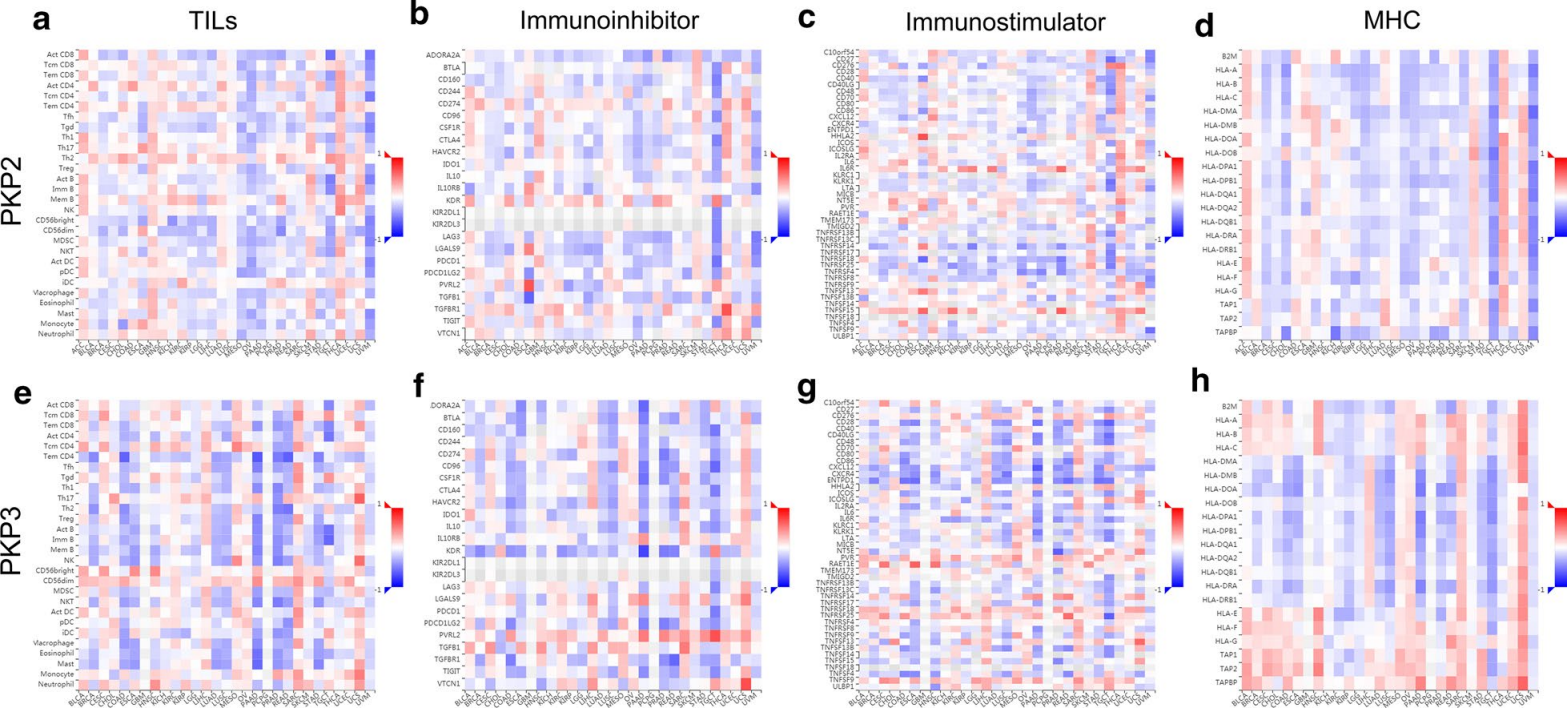
Correlation analysis between PKP2/3 expression and immune molecules.** a–d** The abundances of TILs, immunoinhibitors, immunostimulators, and MHCs correlated with PKP2 expression. **e–h** The abundances of TILs, immunoinhibitors, immunostimulators, and MHCs correlated with PKP3 expression. Positive and negative correlations colored by red and blue, respectively. The color intensity is proportional to correlations. *TILs* tumor-infiltrating lymphocytes, *MHC* major histocompatibility

The relationships between the expression of PKP2/3 and tumor-infiltrating lymphocytes (TILs) and immunomodulators were analyzed by Spearman correlation using TISIDB database (Fig. 7). The results indicated that there was no significant positive correlation between TILs, immunoinhibitors, MHCs and PKP2 (Fig. 7a, b and d). Figure 7c and Additional file 1: Figure S1a, b showed the immunostimulators displaying the greatest correlation with PKP2 included HHLA2 (rho = 0.177, *P* = 1.93e−03) and TNFRSF13C (rho = 0.117, *P* = 0.0411). Figure 7e–h showed that there was a correlation between the expression of PKP3 and TILs, immunoinhibitors, immunostimulators, MHCs. According to Additional file 1: Figure S2a–c, the TILs showing the strongest correlation with PKP3 included Tcm_CD4 (rho = 0.256, *P* = 5.78e−06), pDC (rho = 0.232, *P* = 4.12e−05) and MDSC (rho = 0.158, *P* = 5.55e−03). The immunoinhibitors displaying the strongest correlation with PKP3 included LGALS9 (rho = 0.312, *P* = 2.73e−08), VTCN1 (rho = 0.185, *P* = 1.18e−03) and IL10RB (rho = 0.18, *P* = 1.56e−03) (Additional file 1: Figure S2d–f). The immunostimulators showing the greatest correlation with PKP3 included TNFRSF14 (rho = 0.334, *P* = 2.44e−09), C10orf54 (rho = 0.291, *P* = 2.42e−07) and TMEM173 (rho = 0.258, *P* = 5.02e−06) (Additional file 1: Figure S3g–i). The MHCs displaying the greatest correlation with PKP3 included TAP2 (rho = 0.262, *P* = 3.55e−06), HLA-DRB1 (rho = 0.194, *P* = 6.29 l-04) and TAPBP (rho = 0.19, *P* = 8.55e−04) (Additional file 1: Figure S2j–l).

### High PKP2 expression in ovarian cancer validated by IHC

**Fig. 8:**
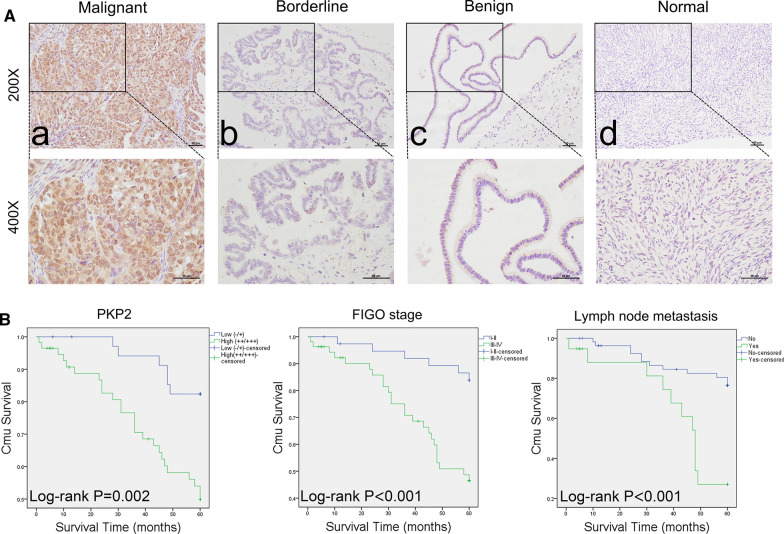
Expression and the prognosis value of PKP2 in ovarian cancer.** A** Immunohistochemical staining of PKP2 in malignant tissues (**a**), borderline tissues (**b**), benign tissues (**c**), and normal tissues (**d**). **B** Relationship between PKP2 expression, FIGO stage, lymph node metastasis and prognosis of patients with ovarian cancer

Immunohistochemistry showed that PKP2 was mainly expressed in cell nucleus, cytoplasm and membrane in ovarian cancer tissues (Fig. 8A (a)). The expression of PKP2 in mucinous carcinoma, endometrioid carcinoma and clear cell carcinoma was also displayed in Additional file 1: Figure S3a–c. The positive expression rate of PKP2 was 83.8% (98/117) in ovarian cancer, which was significantly higher than those in borderline group (43.5%, 10/23), benign group (26.7%, 4/15) and normal group (20%, 3/15) (all *P* < 0.001) (Fig. 8A (b–d), Table 2). The positive expression rates of PKP2 in borderline group and benign group were also higher than that in normal tissue, but the differences were not statistically significant (both *P* > 0.05). We further divided different ovarian tissues into low (−/ +) and high PKP2 expression group (+ + / +  + +) (Additional file 1: Figure S4a–d). The results showed that the high positive expression rate of PKP2 in malignant group was 62.4% (73/117), which was significantly higher than that in borderline group (34.8%, 8/23), benign group (20%, 3/15) and normal group (13.3%, 2/15) (all *P* < 0.05), and the high positive expression rate of PKP2 in borderline group was also significantly higher than that in normal group (*P* < 0.05) (Fig. 8A, Table 2). The images of negative and positive controls of PKP2 expression with IHC were displayed in Additional file 1: Figure S5.

**Table 2:** Expression of PKP2 in different ovarian tissues

Groups	Cases	Low		High		Positive rate (%)	High expression rate (%)
		**−**	** + **	** + + **	** + + + **		
Malignant	117	19	25	48	25	83.8^a,b,c^	62.4^d,e,f^
Borderline	23	13	2	4	4	43.5	34.8^g^
Benign	15	11	1	1	2	26.7	20.0
Normal	15	12	1	2	0	20.0	13.3

^a,b,c^Indicated that the positive expression rate of PKP2 in malignant tissues was compared with that in borderline group, benign group and normal tissues, all *P*<0.05 (*P*_a_<0.001, *P*_b_<0.001, *P*_c_<0.001); ^d,e,f^Indicated that the high positive rate of PKP2 in malignant tissues is higher than that in borderline group, benign group and normal tissues, all *P*<0.05 (*P*_d_ = 0.014, *P*_e_ = 002, *P*_e_<0.001); ^g^Indicated that the high positive expression rate of PKP2 in borderline tissues was compared with that in normal tissues, *P*<0.05(*P*_g_ = 0.030)

### The relationship between the expression of PKP2 and clinicopathological parameters of ovarian cancer

A total of 117 cases of ovarian cancer tissues were divided into low (−/ +) and high (+ + / +  + +) PKP2 expression groups. The positive expression rate of PKP2 was 92.5% (62/67) in patients with FIGO stage III-IV, which was higher than those in stage I-II (72%, 36/50) (*P* < 0.05). The positive expression rate of PKP2 in poor differentiated group (92.5%, 49/53) was also significantly higher than that in well-moderate differentiated group (76.6%, 49/64) (*P* < 0.05). There were no statistically differences in PKP2 expression between lymph node metastasis group (91.7%, 22/24) and non-metastasis group (78.6%, 55/70) (*P* > 0.05). Furthermore, the high positive expression rate of PKP2 in patients with stages III ~ IV (70.1%, 47/67) was also higher than that in stages I ~ II (52.0%, 26/50) (*P* < 0.05). No significant differences were detected between the expression of PKP2 and the age, pathological type of ovarian cancer (*P* > 0.05) (Table 3).

**Table 3:** Relationships between the expression of PKP2 and clinicopathological parameters of 117 ovarian cancer patients

Groups	Cases	Low		High		Positive rate (%)	*P *value	High expression rate (%)	*P *value
		(−)	( +)	(+ +)	(+ + +)				
Age at diagnosis									
< 59	74	13	15	31	15	82.4	*P* = 0.744	62.2	*P* = 0.946
≥59	43	6	10	17	10	86.0		62.8	
Pathological type									
Serous	76	11	14	37	14	85.5	*P* = 0.106	61.7	*P* = 0.144
Mucinous	13	2	3	5	3	84.6		61.5	
Endometrioid	20	6	6	4	4	70.0		40.0	
Clear cell carcinoma	8	0	2	2	4	100.0		75.0	
FIGO stage									
I-II	50	14	10	16	10	72.0	*P* = 0.006	52.0	*P* = 0.045
III-IV	67	5	15	32	15	92.5		70.1	
Differentiation									
Well	35	8	9	11	7	77.1	*P*well-mod vs. poor = 0.033	51.4	*P*well-mod vs. poor = 0.132
Moderate	29	7	4	13	5	75.9		62.1	
Poor	53	4	12	24	13	92.5		69.8	
Lymphatic metastasis									
No	70	15	16	25	14	78.6	*P* = 0.318	55.7	*P* = 0.095
Yes	24	2	4	11	7	91.7		75.0	
Unknown	23	2	5	12	4	91.3		69.6	

### High expression of PKP2 was an independent risk factor associated with poor prognosis in patients with ovarian cancer

**Fig. 9:**
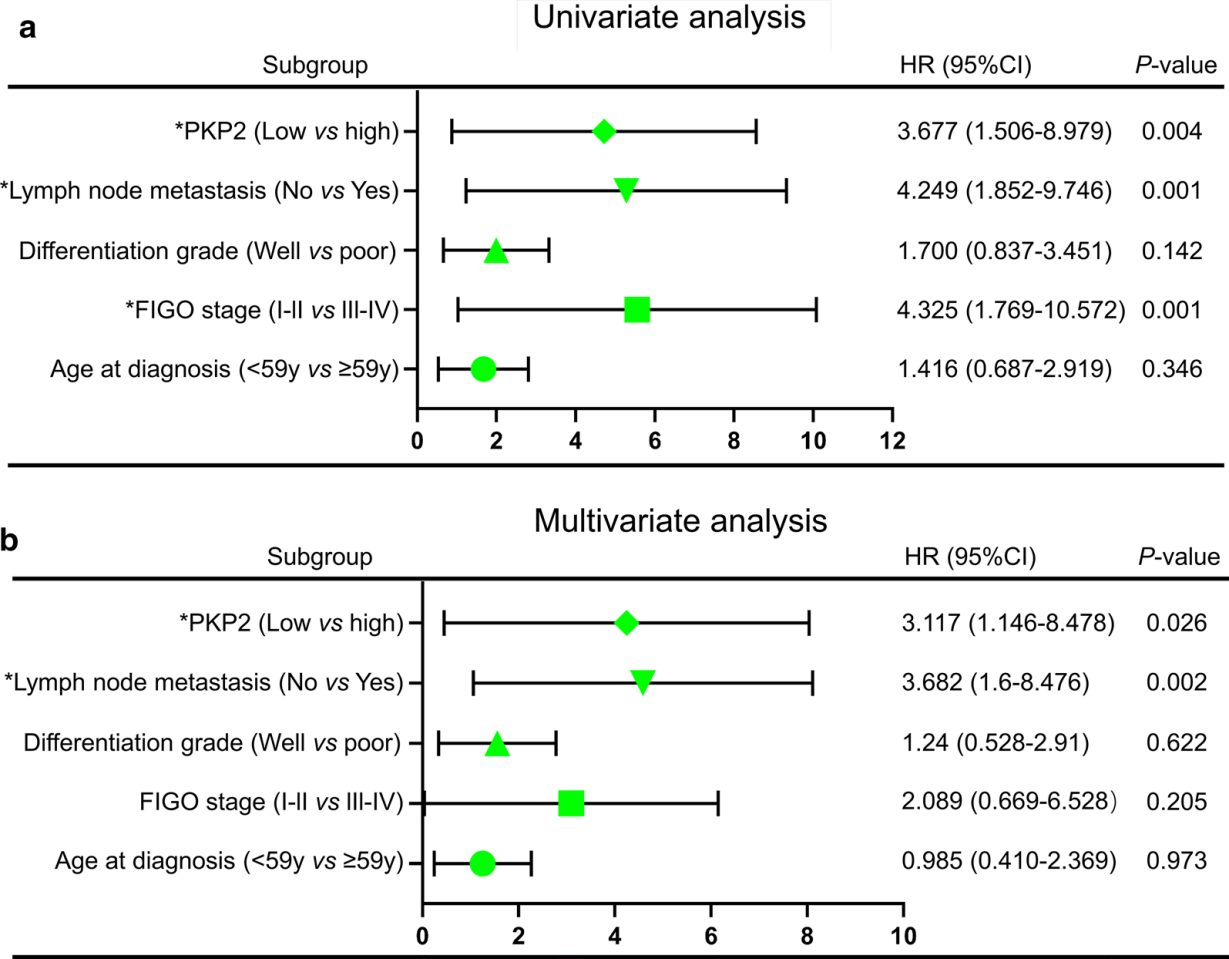
Univariate (**a**) and multivariate (**b**) Cox regression analysis of overall survival of patients with ovarian cancer by forest map

We further explored the correlation of PKP2 expression with the prognosis in ovarian cancer. As of April 1, 2019, 117 patients with ovarian cancer were followed up, 25 patients were lost and 31 patients died, the survival time varied from 1 to 123 months. High expression of PKP2 was significantly associated with poor prognosis (Fig. 8c). Furthermore, FIGO stage (I- II vs. III-IV) and lymph node metastasis (No *vs* Yes) were both correlated with poor prognosis in ovarian cancer (all *P* < 0.05) (Fig. 8c). Cox regression model was performed to analyze the relationships between the prognosis and clinicopathological parameters in ovarian cancer. Univariate and multivariate analysis showed that high PKP2 expression (HR = 3.117, 95% CI = 1.146–8.478, *P* = 0.026) and lymph node metastasis (HR = 3.682, 95% CI = 1.6–8.476, *P* = 0.002) were independent risk factors for prognosis of patients with ovarian cancer. Forest maps were adopted to visualize univariate and multivariate Cox regression analysis (Fig. 9a, b).

## Discussion

Plakophilins (PKP) are a multifunctional protein family that play a central role in the regulation of cell–cell junction, cytoskeleton reconstruction and vesicle trafficking. In recent years, the role of PKPs in the invasion and metastasis of malignant tumors has gradually attracted the attention of many experts, and the function of PKP1/2/3 has been preliminarily validated in the occurrence and development of many malignant tumors [12]. However, the expression and molecular mechanisms of PKP1/2/3 in ovarian cancer remain undefined. Therefore, it is meaningful to investigate potential role and molecular mechanisms of PKP1/2/3 in the development and progression of ovarian cancer.

As a member of the armadillo family, PKP1 could enhance the recruitment of endogenous desmosome proteins and maintain the stability and integrity of desmosomes [27, 28]. It was reported PKP1 could directly bind to eukaryotic translation initiation factor elF4Al and promoted mRNA translation [29]. Based on the function of PKP1 in protein translation, researchers found that PKP1 could regulate cell growth and proliferation, thus affecting tumor progression and invasion. Many studies have suggested that the expression of PKP1 was significantly decreased or deleted in malignant tumors, including oral/pharyngeal squamous cell carcinoma, esophageal squamous cell carcinoma, prostate cancer [30–32], and high expression of PKP1 inhibited proliferation, migration and invasion of these tumors, suggesting that PKP1 may be served as a tumor suppressor gene. Recent studies have shown that knockout of PKP1 by CRISPR-Cas9 technique significantly inhibited tumor proliferation and promoted metastasis and dissemination of lung cancer, and PKP1 promoted MYC translation by binding 5′-UTR of MYC mRNA, suggesting that PKP1 was also recognized as an oncogene in lung cancer [33]. In this study, we found that the levels of PKP1 mRNA and protein were not significantly upregulated in ovarian cancer, indicating that PKP1 may not exert valuable function in occurrence and development of ovarian cancer, but the research need to be further verified by larger clinical samples and experimental evidence.

PKP2, as a characteristic desmosome plaque protein, is mainly expressed in monolayer or stratified epithelial cells and non-epithelial tissues such as myocardium and lymph node follicles, and is the only PKP expressed in myocardium and liver [34, 35], which can stabilize desmosome cadherin on the cell membrane. As early as the discovery of PKP2, researchers found that the expression level of PKP2 was significant different in tumors with varying degrees of differentiation [36]. High expression of PKP2 was also associated with the prognosis and promoted proliferation and invasion in gliomas and lung adenocarcinomas [37, 38], suggesting that PKP2 could promote tumor progression. However, many studies have showed that PKP2 was decreased in some malignant tumors, such as gastric cancer and bladder cancer [39, 40], and negatively correlated with the prognosis, proliferation and invasion of these tumors, indicating that PKP2 can also act as a tumor suppressor gene in malignant tumors. The oncogene or anti-oncogene role of PKP2 in tumors mainly dependent on tissue types, but its mechanism still remained unclear. In this study, we found that the expression of PKP2 was overexpressed and significantly correlated with PFS in ovarian cancer with bioinformatics and further validated that PKP2 was stained in cell nucleus, membrane and cytoplasm, and high expression of PKP2 was associated with advanced FIGO stages and differentiation degree by immunohistochemistry. Moreover, high expression of PKP2 was an independent risk factor affecting the survival and prognosis in ovarian cancer, suggesting that PKP2 may play a crucial role in occurrence, development and prognosis in ovarian cancer, and its potential mechanism needs to be further explored and validated.

PKP3, as the most widely expressed member of the PKP family, is commonly expressed in monolayer and stratified epithelial tissues containing desmosomes except for hepatocytes and cardio myocytes [41, 42]. It’s suggested that PKP3 participated in RNA metabolism, gene post-transcriptional regulation and protein synthesis of tumor cells. In recent years, the function and mechanism of PKP3 in tumorigenesis and development were controversial. Many researchers have found that the expression of PKP3 was significantly increased in non-small cell lung cancer and prostate cancer, and associated with the prognosis and progression of tumors [32, 43]. Valladares et al*.* showed that the overexpression of PKP3 mRNA in the blood was of great value for the early diagnosis of gastrointestinal tumor [44], suggesting that PKP3 could play a vital role in carcinogenesis and prognosis of tumor. However, some studies have shown that PKP3 was significantly downregulated in gastric cancer and bladder cancer, and downregulation of PKP3 promoted the progression and invasion of tumors [39, 40]. Above studies suggested that the role of PKP3 in malignant tumors may depend on tissue types, cell types, differentiation degree and potential signal pathway. In this study, we found that the expression of PKP3 mRNA and protein was upregulated in ovarian cancer and significantly correlated with PFS in patients with ovarian cancer. Qian et al. showed that PKP3 was highly expressed in both cytoplasm and nucleus of ovarian cancer, which was consistent with our research, high expression of PKP3 was correlated with advanced FIGO stage, lymph node metastasis, and poor prognosis [13]. Lim et al*.* confirmed that PKP3 could regulate proliferation, invasion and autophagy via MAPK-JNK-ERK1/2-mTOR pathway in ovarian cancer [14]. It is enough to prove that PKP3 plays a crucial role in prognosis evaluation and potential therapeutic target of ovarian cancer, but the specific mechanism remains to be further explored.

In order to further clarify copy number variation of PKP1/2/3 in ovarian cancer, cBioPortal database was adopted to calculate the percentages of gene variation of PKP1/2/3 in ovarian cancer. It was found that the incidence of amplifications in PKP2 was 6.27%, and PKP2 was related to the survival of patients with ovarian cancer, suggesting that the increase of gene amplification may be correlated with the prognosis of tumor. We further constructed a gene co-expression network with PKPs and their top 50 neighbor genes, suggesting that PKPs may exert their function by interacting with proteins. De S et al*.* have reported that 14-3-3 (YWHA) proteins, which have seven isoforms of 14-3-3 and were identified in the ovary [45–48], are known to interact with and regulate several binding partners including CDC25B phosphatase and PKPs [49–51]. It is of great value to explore the underlying mechanism of the interaction between YWHA and PKPs in ovarian cancer. Researchers found that PKP1 interacted with RNA-binding proteins such as G3BP, and inhibited tumorigenesis and development by regulating the post-transcription of the gene [36]. The N-terminal domain of PKP2 could directly bind to epidermal growth factor receptor (EGFR) and positively regulate the signal transduction of EGFR [52]. In addition, PKP3 could closely bind to RNA-binding proteins such as FXR1 and PABPC1 [53], indicating that PKP1/2/3 can participate in cell connection and tumor progression through its interacting proteins. According to the above research, our study indicated that the expression level and prognostic value of PKP1 showed little significance in ovarian cancer, and PKP2/3 may exert profound influence in the progression of ovarian cancer. We only explored the regulatory network, functional mechanism and immune infiltration of PKP2/3 in OC subsequently. In terms of potential mechanism, PKP2 could regulate the activity of Wnt/β-catenin in colon cancer-associated fibroblasts [54], and PKP2 promoted the proliferation and migration of lung cancer by activating EGFR signaling pathway [38]. In addition, PKP3 regulated autophagy and invasion of ovarian cancer through MAPK/JNK/ERK1/2/mTOR signaling pathway [13, 14]. GO and KEGG enrichment analysis suggested that PKP2/3 and their correlated genes were mainly involved in the formation of plasma membrane, collagen-containing extracellular matrix, cell–cell and anchoring junction. These genes exerted their biological functions by binding to various proteins, such as proteoglycan, protein domain, adhesion molecule and actin. What’s more, PKP2/3 and their closely related genes participated in cytokine-mediated signaling pathway, receptor signaling pathway, pathways in cancer and other signal pathways. The above signaling pathways could participate in the progression and biological behaviors of ovarian cancer [55]. This study provides powerful evidences that PKP2/3 may affect the occurrence, development and biological behavior of ovarian cancer by regulating interacting proteins and signaling pathways.

Ovarian cancer possesses the characteristic of immunogenicity and can be recognized and attacked by the immune system, which plays a well-established role in the pathogenesis and progression of ovarian cancer. Accumulating studies have indicated that immune infiltration affected the survival, prognosis and immunotherapy of ovarian cancer. In recent years, immunotherapy has also become auxiliary therapeutic treatment for advanced and recurrent ovarian cancer, including a variety of vaccines, monoconal antibodies (bevacizumab, Cediranib), immune checkpoint inhibitors for PD1/PDL1 axis and related cytokines [56]. In this study, we found that PKP2 was also associated with immunostimulators (HHLA2 and TNFRSF13C). There was a greatest correlation between PKP3 and lymphocytes (Tcm_CD4, pDC, and MDSC), immunoinhibitors (LGALS9, VTCN1, and IL10RB), immunostimulators (TNFRSF14, C10orf54, and TMEM173), and MHCs (TAP2, HLA-DRB1, and TAPBP). Researchers found that PKP2 could be detected in immune cells such as progenitor cells, specific T-cells dendritic cells, and basophils [57], and PKP3 was upregulated in neutrophils after stimulated by LPS or PMA, and played a vital role in both local and systemic induced immune responses [58]. The results suggested that PKP2/3 may affect the survival and prognosis of ovarian cancer by regulating the immune infiltration in tumor microenvironment, which can provide new directions and novel approach for immunotherapy of ovarian cancer.

However, due to limitations of online database and bioinformatics tools, additional experimental validations in vivo and vitro are still required to further explore the functional mechanisms of PKP2/3, and the role of PKP2/3 in therapeutic targets should be investigated and verified. In the future, based on the present research, we will conduct detailed and comprehensive studies to provide more powerful evidence to support our findings.

## Conclusion

In summary, with various bioinformatics tools, integrated databases and immunohistochemistry, our study provided sufficient evidences that overexpression of PKP2/3 were closely correlated with PFS and poor prognosis in OC, and genomic alteration and co-expression gene of PKP2/3 were also illuminated. We further identified that PKP2/3 could participate in a variety of biological functions, signaling pathways and immune infiltration, which provided novel and valuable insights into the molecular mechanisms underlying the initiation and progression of ovarian cancer. Therefore, PKP2/3 can potentially be identified as tumor biomarkers for early diagnosis and prognosis evaluation of ovarian cancer.

## Supplementary information

**Additional file 1: Table S1. **The expression of PKP1/2/3 protein in ovarian cancer and normal tissues by immunohistochemistry (Human Protein Atlas). **Table S2. **The Prognostic values of PKP1/2/3 in all and P53 mutated patients with OC (Kaplan-Meier plotter). **Table S3. **Significantly enriched GO (Cellular Components) analysis of PKP2 and co-expression genes in OV (Metascape). **Table S4. **Significantly enriched GO (Molecular Function) analysis of PKP2 and co-expression genes in OV (Metascape). **Table S5. **Significantly enriched GO (Biological Processes) analysis of PKP2 and co-expression genes in OV (Metascape). **Table S6. **Significantly enriched KEGG pathway analysis of PKP2 and co-expression genes in OV (Metascape). **Table S7. **Significantly enriched GO (Cellular Components) analysis of PKP3 and co-expression genes in OV (Metascape). **Table S8. **Significantly enriched GO (Molecular Function) analysis of PKP3 and co-expression genes in OV (Metascape). **Table S9. **Significantly enriched GO (Biological Processes) analysis of PKP3 and co-expression genes in OV (Metascape). **Table S10. **Significantly enriched KEGG pathway analysis of PKP3 and co-expression genes in OV (Metascape). **Table S11. **Expression of PKP2 in different ovarian tissues including 53 high grade ovarian cancer. **Table S12. **Relationships between the expression of PKP2 and clinicopathological parameters of 53 high grade. **Figure S1. **Relationship between PKP2 expression and immunostimulators in OV (TISIDB). **Figure S2. **Relationship between PKP3 expression and TILs, immunomodulators in OV (TISIDB). **Figure S3. **Expression of PKP2 in different ovarian cancer tissues. **Figure S4. **High expression of PKP2 in different ovarian tissues. **Figure S5. **The negative and positive control of PKP2 expression by IHC.

## Data Availability

The datasets used or analyzed during the current study are available from the corresponding author upon reasonable request.

## Abbreviations

OC: Ovarian cancer
OV: Ovarian serous cystadenocarcinoma
PKP: Plakophilin
ARVC: Arrhythmogenic right ventricular cardiomyopathy
TIMER: Tumor Immune Estimation Resource
GEPIA: Gene Expression Profiling Interactive Analysis
HPA: Human Protein Atlas
HR: Hazard ratio
CI: Confidence interval
OS: Overall survival
PFS: Progression-free survival
CNA: DNA copy number change
PPI: Protein–protein interaction
FDR: False discovery rate
TCGA: The Cancer Genome Atlas
GO: Gene Ontology
KEGG: Kyoto Encyclopedia of Genes and Genomes
SP: Streptavidin-peroxidase
FITC: Fluorescein isothiocyanate
DAPI: 4,6-Diamidino-2-phenylindole
IHC: Immunohistochemistry
FIGO: The International Federation of Gynecology and Obstetrics
