# Supplementary material for: Identification of PKP 2/3 as potential biomarkers of ovarian cancer based on bioinformatics and experiments
Source: Cancer Cell Int. 2020 Oct 17;20:509. doi: 10.1186/s12935-020-01602-3 (PMC7568375; doi:10.1186/s12935-020-01602-3)
Supplement: Supplementary file 1 — Additional file 1: Table S1. The expression of PKP1/2/3 protein in ovarian cancer and normal tissues by immunohistochemistry (Human Protein Atlas). Table S2. The Prognostic values of PKP1/2/3 in all and P53 mutated patients with OC (Kaplan-Meier plotter). Table S3. Significantly enriched GO (Cellular Components) analysis of PKP2 and co-expression genes in OV (Metascape). Table S4. Significantly enriched GO (Molecular Function) analysis of PKP2 and co-expression genes in OV (Metascape). Table S5. Significantly enriched GO (Biological Processes) analysis of PKP2 and co-expression genes in OV (Metascape). Table S6. Significantly enriched KEGG pathway analysis of PKP2 and co-expression genes in OV (Metascape). Table S7. Significantly enriched GO (Cellular Components) analysis of PKP3 and co-expression genes in OV (Metascape). Table S8. Significantly enriched GO (Molecular Function) analysis of PKP3 and co-expression genes in OV (Metascape). Table S9. Significantly enriched GO (Biological Processes) analysis of PKP3 and co-expression genes in OV (Metascape). Table S10. Significantly enriched KEGG pathway analysis of PKP3 and co-expression genes in OV (Metascape). Table S11. Expression of PKP2 in different ovarian tissues including 53 high grade ovarian cancer. Table S12. Relationships between the expression of PKP2 and clinicopathological parameters of 53 high grade. Figure S1. Relationship between PKP2 expression and immunostimulators in OV (TISIDB). Figure S2. Relationship between PKP3 expression and TILs, immunomodulators in OV (TISIDB). Figure S3. Expression of PKP2 in different ovarian cancer tissues. Figure S4. High expression of PKP2 in different ovarian tissues. Figure S5. The negative and positive control of PKP2 expression by IHC. [file 12935_2020_1602_MOESM1_ESM.docx]

**
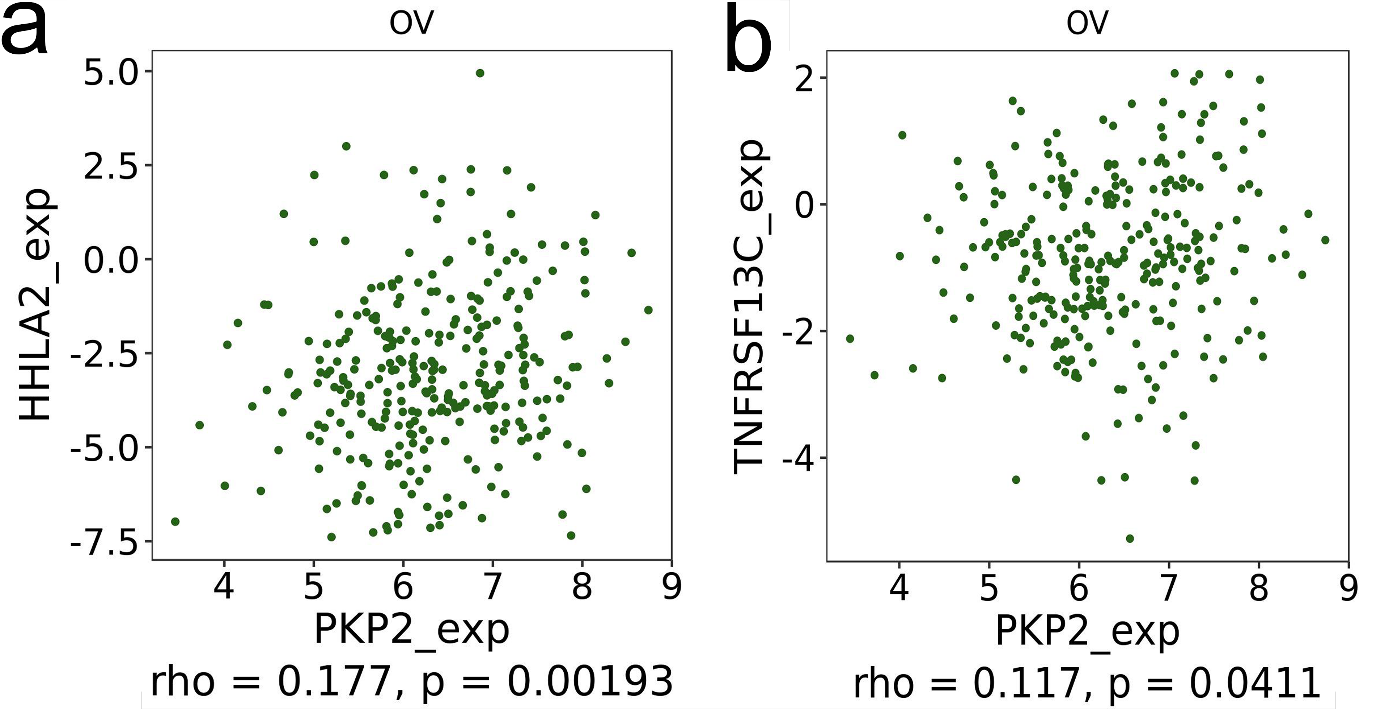
**

**Figure S1 Relationship between PKP2 expression and immunostimulators in OV (TISIDB).**

**a-b** The Spearman’s correlation of PKP2 expression with immunostimulators (TOP 2) with scatter plot.


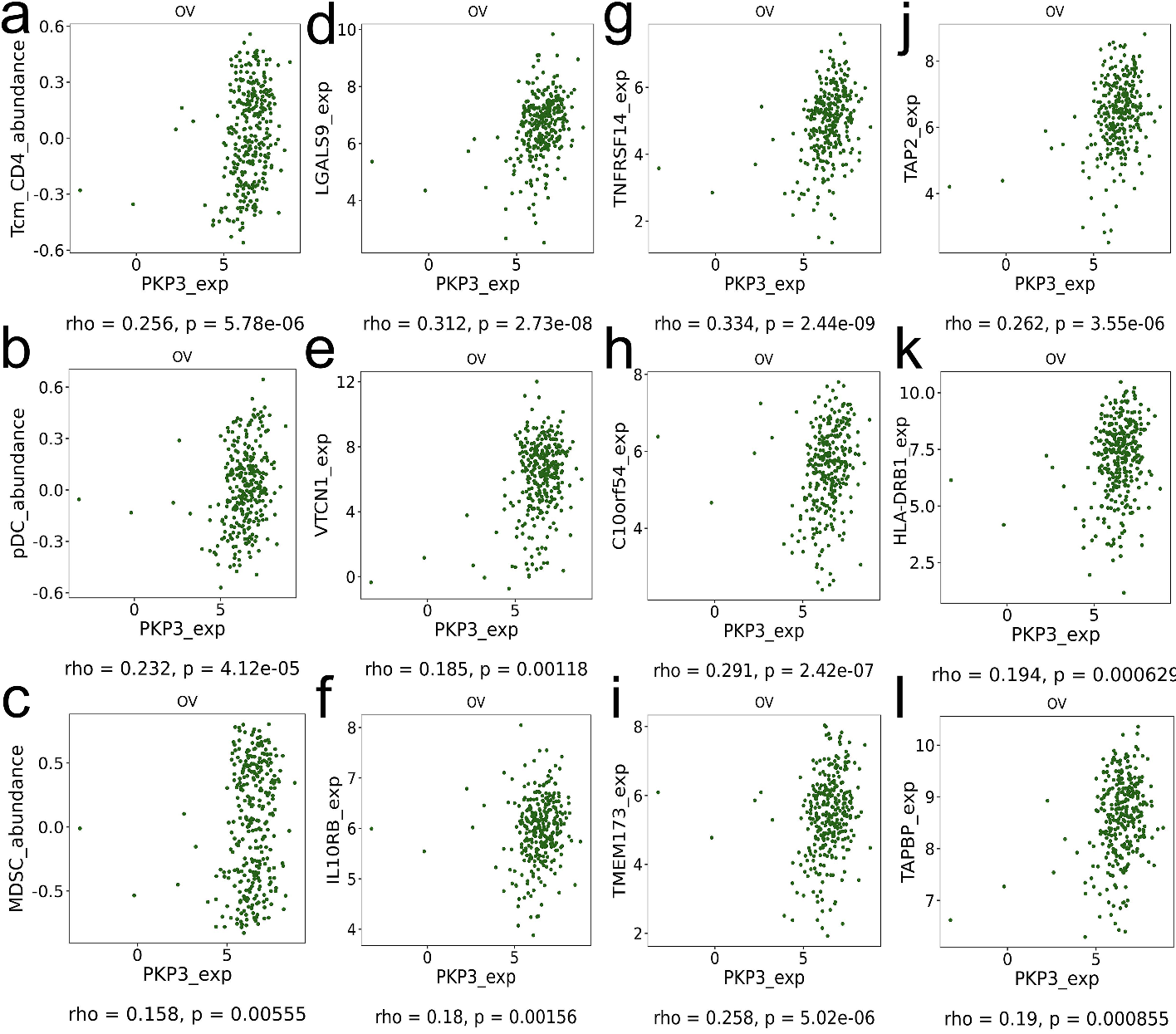


**Figure S2 Relationship between PKP3 expression and TILs, immunomodulators in OV (TISIDB)**

**a-c** The Spearman’s correlation of PKP3 expression with TILs (TOP 3) with scatter plot. **d-f** The Spearman’s correlation of PKP3 expression with immunoinhibitors (TOP 3) with scatter plot. **g-i** The Spearman’s correlation of PKP3 expression with immunostimulators (TOP 3) with scatter plot. **j-l** The Spearman’s correlation of PKP3 expression with MHCs (TOP 3) with scatter plot. TILs: tumor-infiltrating lymphocytes, MHC: major histocompatibility complex.

**
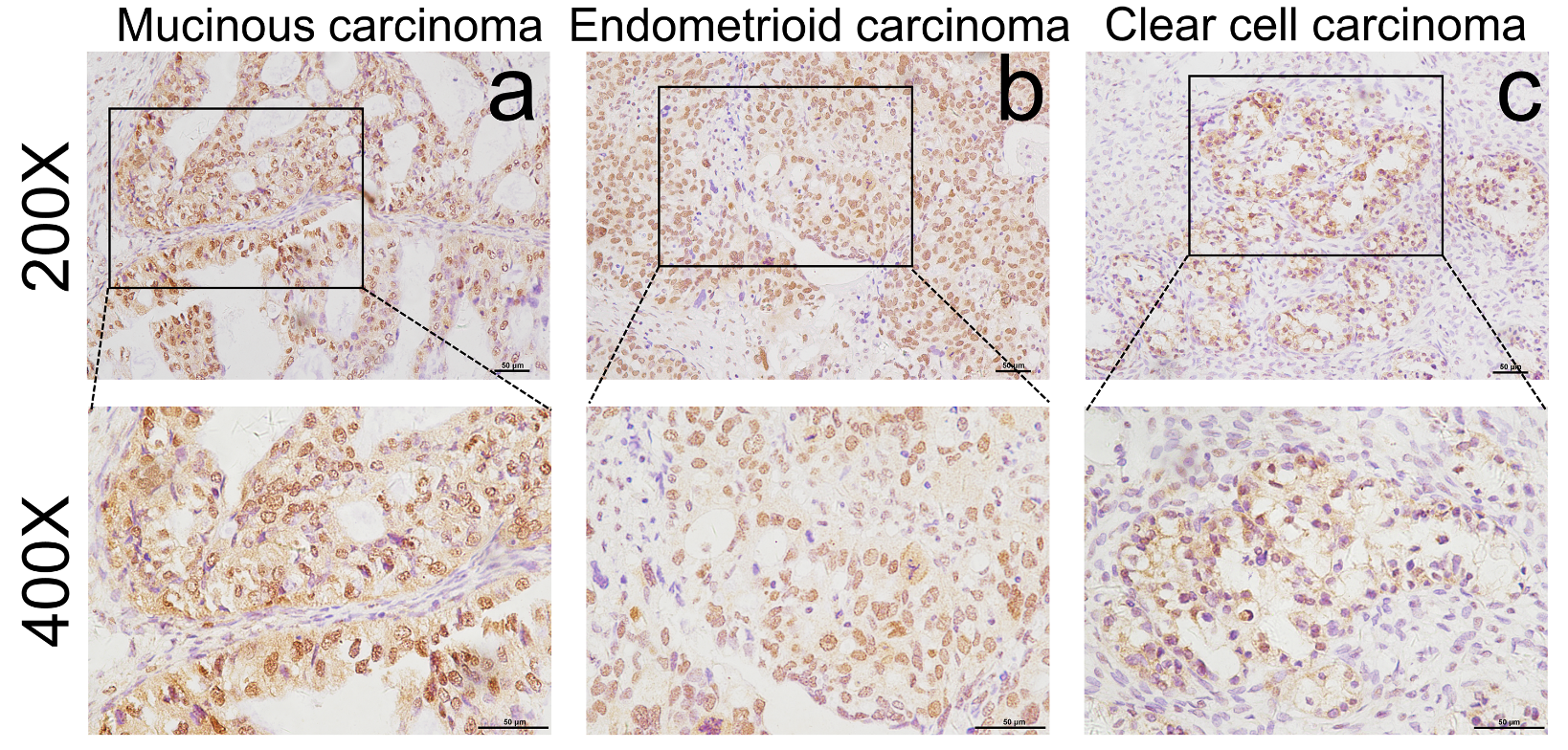
**

**Figure S3 Expression of PKP2 in different ovarian cancer tissues.** Immunohistochemical staining of high expression of PKP2 in mucinous carcinoma (**a**), endometrioid carcinoma (**b**) and clear cell carcinoma(**c**).

**
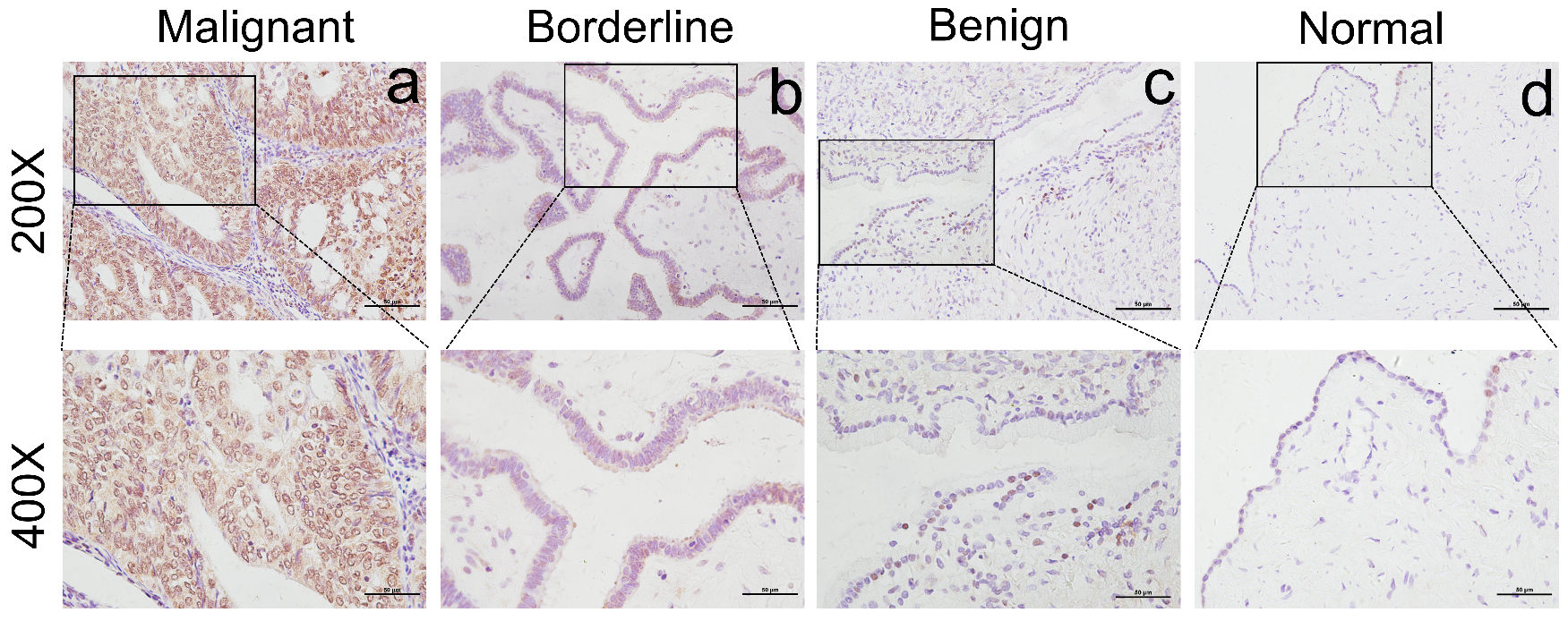
**

**Figure S4 High expression of PKP2 in different ovarian tissues.** Immunohistochemical staining of high expression of PKP2 in malignant tissues (**a**), borderline tissues (**b**), benign tissues (**c**), and normal tissues.


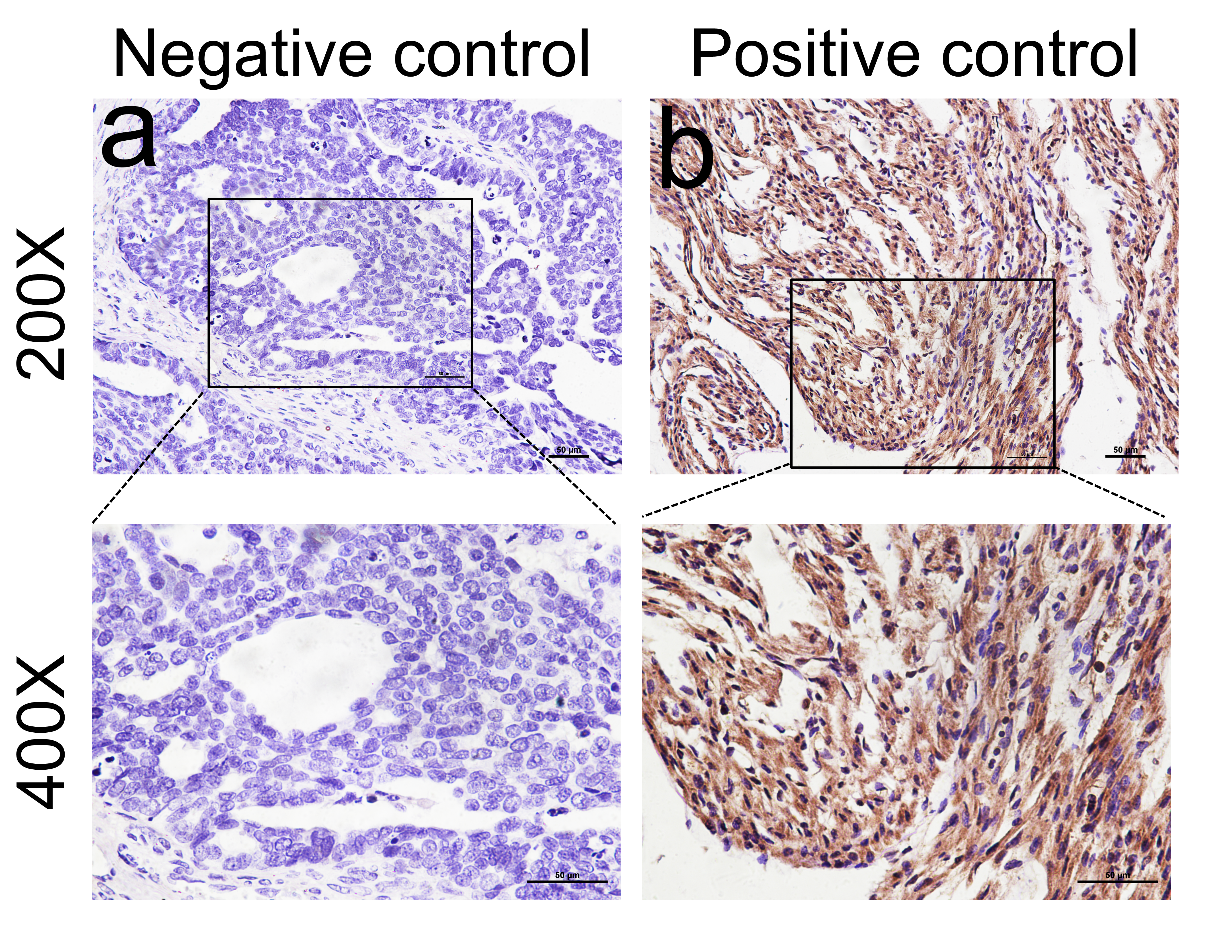


**Figure S5 The negative and positive control of PKP2 expression by IHC. a** Phosphate-buffered saline (PBS) served as a negative control instead of primary antibody with ovarian cancer tissue. **b** Human heart muscle tissue served as positive control. Scale bar: 50um.

**Table S1 The expression of PKP1/2/3 protein in ovarian cancer and normal tissues by** **immunohistochemistry (Human Protein Atlas)**

| **Gene** | **Figure** | **Patient ID** | **Age** | **Tissue** | **Antibody** | **Staining** | **Intensity** | **Quantity**  **%** | **Location** |
| --- | --- | --- | --- | --- | --- | --- | --- | --- | --- |
| PKP1 | 1T | 3116 | 55 | Cystadenocarcinoma, serous | HPA027221 | Not detected | Negative | None | None |
|  | 2T | 3116 | 55 | Cystadenocarcinoma, serous | HPA027221 | Not detected | Negative | None | None |
|  | 1N | 2004 | 27 | Normal tissue | HPA027221 | Not detected | Negative | None | None |
|  | 2N | 2004 | 27 | Normal tissue | HPA027221 | Not detected | Negative | None | None |
| PKP2 | 1T | 3146 | 44 | Cystadenocarcinoma, serous | HPA014314 | Medium | Moderate | >75% | Nuclear/cytoplasmic/  membranous |
|  | 2T | 3146 | 44 | Cystadenocarcinoma, serous | HPA014314 | Medium | Moderate | >75% | Nuclear/cytoplasmic/  membranous |
|  | 1N | 2159 | 33 | Normal tissue | HPA014314 | Not detected | Weak | <25% | Cytoplasmic/membranous |
|  | 2N | 2159 | 33 | Normal tissue | HPA014314 | Not detected | Weak | <25% | Cytoplasmic/membranous |
| PKP3 | 1T | 2982 | 53 | Cystadenocarcinoma, serous | CAB012993 | Medium | Moderate | >75% | Cytoplasmic/membranous |
|  | 2T | 2982 | 53 | Cystadenocarcinoma, serous | CAB012993 | Medium | Moderate | >75% | Cytoplasmic/membranous |
|  | 1N | 2159 | 33 | Normal tissue | CAB012993 | Not detected | Negative | None | None |
|  | 2N | 2159 | 33 | Normal tissue | CAB012993 | Not detected | Negative | None | None |

**Table S2 The Prognostic values of PKP1/2/3 in all and P53 mutated patients with OC (Kaplan-Meier plotter).**

| **PKP family** | **OS** | | |  | **PFS** | | |  | ***TP53* mutation** | | | | | | | |
| --- | --- | --- | --- | --- | --- | --- | --- | --- | --- | --- | --- | --- | --- | --- | --- | --- |
|  |  |  |  |  |  |  |  |  | **OS** | | |  | | **PFS** | | |
|  | **HR** | **95% CI** | ***P*-value** | | **HR** | **95% CI** | ***P*-value** |  | **HR** | **95% CI** | ***P*-value** | | **HR** | | **95% CI** | ***P*-value** |
| PKP1 | 0.86 | 0.75-1 | 0.051 |  | **0.84** | **0.73-0.95** | **0.006** |  | **1.28** | **1.01-1.63** | **0.044** |  | | **1.41** | **1.11-1.79** | **0.0045** |
| PKP2 | 0.91 | 0.79-1.04 | 0.18 |  | **1.19** | **1.04-1.36** | **0.01** |  | 1.21 | 0.96-1.52 | 0.099 |  | | 1.27 | 0.99-1.63 | 0.057 |
| PKP3 | **0.82** | **0.71-0.95** | **0.0075** |  | **0.84** | **0.74-0.95** | **0.0069** |  | **1.34** | **1.05−1.7** | **0.017** |  | | **1.58** | **1.25 − 2** | **0.00014** |

**Table S3 Significantly enriched GO (Cellular Components) analysis of PKP2 and co-expression genes in OV (Metascape).**

| **Term** | **Category** | **Description** | **Log *P*** | **Log (*q*-value)** | **Count** | **%** |
| --- | --- | --- | --- | --- | --- | --- |
| GO:0009897 | GO Cellular Components | external side of plasma membrane | -10.81 | -7.52 | 33 | 8.21 |
| GO:0036021 | GO Cellular Components | endolysosome lumen | -6.07 | -3.26 | 4 | 80.00 |
| GO:0062023 | GO Cellular Components | collagen-containing extracellular matrix | -5.85 | -3.16 | 25 | 6.10 |
| GO:0098797 | GO Cellular Components | plasma membrane protein complex | -5.59 | -3.00 | 34 | 4.92 |
| GO:0030659 | GO Cellular Components | cytoplasmic vesicle membrane | -4.47 | -2.02 | 34 | 4.35 |
| GO:0042105 | GO Cellular Components | alpha-beta T cell receptor complex | -4.08 | -1.81 | 3 | 60.00 |
| GO:0001772 | GO Cellular Components | immunological synapse | -4.02 | -1.81 | 6 | 16.22 |
| GO:0044440 | GO Cellular Components | endosomal part | -3.88 | -1.74 | 25 | 4.66 |
| GO:0015629 | GO Cellular Components | actin cytoskeleton | -3.73 | -1.62 | 24 | 4.64 |
| GO:0005861 | GO Cellular Components | troponin complex | -3.36 | -1.35 | 3 | 37.50 |
| GO:0008385 | GO Cellular Components | IkappaB kinase complex | -3.19 | -1.22 | 3 | 33.33 |
| GO:1902495 | GO Cellular Components | transmembrane transporter complex | -2.99 | -1.06 | 16 | 4.98 |
| GO:0045121 | GO Cellular Components | membrane raft | -2.91 | -1.05 | 16 | 4.89 |
| GO:0042734 | GO Cellular Components | presynaptic membrane | -2.84 | -1.03 | 10 | 6.41 |
| GO:0005911 | GO Cellular Components | cell-cell junction | -2.45 | -0.75 | 17 | 4.28 |
| GO:0044297 | GO Cellular Components | cell body | -2.39 | -0.71 | 22 | 3.82 |
| GO:0030027 | GO Cellular Components | lamellipodium | -2.12 | -0.50 | 10 | 5.08 |
| GO:0098644 | GO Cellular Components | complex of collagen trimers | -2.07 | -0.46 | 3 | 14.29 |

**Table S4 Significantly enriched GO (Molecular Function) analysis of PKP2 and co-expression genes in OV (Metascape).**

| **Term** | **Category** | **Description** | **Log *P*** | **Log(*q*-value)** | **Count** | **%** |
| --- | --- | --- | --- | --- | --- | --- |
| GO:0004896 | GO Molecular Functions | cytokine receptor activity | -7.04 | -3.37 | 13 | 13.40 |
| GO:0043394 | GO Molecular Functions | proteoglycan binding | -5.15 | -1.88 | 7 | 19.44 |
| GO:0042010 | GO Molecular Functions | interleukin-15 receptor activity | -5.07 | -1.88 | 3 | 100 |
| GO:0004175 | GO Molecular Functions | endopeptidase activity | -4.09 | -1.06 | 22 | 5.14 |
| GO:0019904 | GO Molecular Functions | protein domain specific binding | -3.77 | -0.88 | 30 | 4.21 |
| GO:0001664 | GO Molecular Functions | G protein-coupled receptor binding | -3.51 | -0.77 | 16 | 5.57 |
| GO:0032549 | GO Molecular Functions | ribonucleoside binding | -3.40 | -0.77 | 19 | 4.95 |
| GO:0042803 | GO Molecular Functions | protein homodimerization activity | -3.39 | -0.77 | 27 | 4.18 |
| GO:0008157 | GO Molecular Functions | protein phosphatase 1 binding | -3.19 | -0.73 | 4 | 20.00 |
| GO:0042169 | GO Molecular Functions | SH2 domain binding | -3.05 | -0.70 | 5 | 13.51 |
| GO:0019864 | GO Molecular Functions | IgG binding | -2.91 | -0.67 | 3 | 27.27 |
| GO:0008047 | GO Molecular Functions | enzyme activator activity | -2.90 | -0.67 | 22 | 4.21 |
| GO:0000987 | GO Molecular Functions | proximal promoter sequence-specific DNA binding | -2.88 | -0.67 | 25 | 3.97 |
| GO:0003727 | GO Molecular Functions | single-stranded RNA binding | -2.72 | -0.63 | 7 | 8.14 |
| GO:0140161 | GO Molecular Functions | monocarboxylate:sodium symporter activity | -2.68 | -0.61 | 3 | 23.08 |
| GO:0060090 | GO Molecular Functions | molecular adaptor activity | -2.60 | -0.54 | 13 | 5.08 |
| GO:0005523 | GO Molecular Functions | tropomyosin binding | -2.49 | -0.47 | 3 | 20.00 |
| GO:0008081 | GO Molecular Functions | phosphoric diester hydrolase activity | -2.47 | -0.46 | 7 | 7.37 |
| GO:0003779 | GO Molecular Functions | actin binding | -2.44 | -0.44 | 18 | 4.18 |
| GO:0004675 | GO Molecular Functions | transmembrane receptor protein serine/threonine kinase activity | -2.33 | -0.35 | 3 | 17.65 |

Abbreviations: PKP: plakophilin; GO: Gene Ontology; OV: Ovarian serous cystadenocarcinoma.

**Table S5 Significantly enriched GO (Biological Processes) analysis of PKP2 and co-expression genes in OV (Metascape).**

| **Term** | **Category** | **Description** | **Log *P*** | **Log(*q*-value)** | **Count** | **%** |
| --- | --- | --- | --- | --- | --- | --- |
| GO:0046649 | GO Biological Processes | lymphocyte activation | -20.94 | -16.75 | 63 | 8.42 |
| GO:0002250 | GO Biological Processes | adaptive immune response | -15.10 | -11.60 | 52 | 7.47 |
| GO:0019221 | GO Biological Processes | cytokine-mediated signaling pathway | -11.65 | -8.60 | 50 | 6.28 |
| GO:0030098 | GO Biological Processes | lymphocyte differentiation | -10.87 | -7.93 | 31 | 8.73 |
| GO:0002274 | GO Biological Processes | myeloid leukocyte activation | -8.50 | -5.86 | 39 | 5.95 |
| GO:0019882 | GO Biological Processes | antigen processing and presentation | -7.79 | -5.27 | 21 | 9.01 |
| GO:0033632 | GO Biological Processes | regulation of cell-cell adhesion mediated by integrin | -7.52 | -5.00 | 6 | 54.55 |
| GO:0032496 | GO Biological Processes | response to lipopolysaccharide | -7.43 | -4.92 | 25 | 7.40 |
| GO:0060326 | GO Biological Processes | cell chemotaxis | -7.06 | -4.57 | 23 | 7.57 |
| GO:0006968 | GO Biological Processes | cellular defense response | -6.98 | -4.52 | 10 | 18.87 |
| GO:0031295 | GO Biological Processes | T cell costimulation | -6.75 | -4.31 | 10 | 17.86 |
| GO:0045088 | GO Biological Processes | regulation of innate immune response | -6.64 | -4.24 | 28 | 6.21 |
| GO:0002764 | GO Biological Processes | immune response-regulating signaling pathway | -6.62 | -4.23 | 36 | 5.29 |
| GO:0032623 | GO Biological Processes | interleukin-2 production | -6.18 | -3.85 | 10 | 15.63 |
| GO:0006909 | GO Biological Processes | phagocytosis | -6.07 | -3.78 | 24 | 6.45 |
| GO:0032653 | GO Biological Processes | regulation of interleukin-10 production | -6.01 | -3.73 | 9 | 17.31 |
| GO:0001773 | GO Biological Processes | myeloid dendritic cell activation | -5.93 | -3.68 | 7 | 25.00 |
| GO:0019722 | GO Biological Processes | calcium-mediated signaling | -5.44 | -3.26 | 17 | 7.66 |
| GO:0050777 | GO Biological Processes | negative regulation of immune response | -5.38 | -3.21 | 14 | 8.97 |
| GO:0030574 | GO Biological Processes | collagen catabolic process | -5.35 | -3.19 | 8 | 17.02 |

Abbreviations: PKP: plakophilin; GO: Gene Ontology; OV: Ovarian serous cystadenocarcinoma.

**Table S6 Significantly enriched KEGG pathway analysis of PKP2 and co-expression genes in OV (Metascape).**

| **Term** | **Category** | **Description** | **Log *P*** | **Log(*q*-value)** | **Count** | **%** |
| --- | --- | --- | --- | --- | --- | --- |
| hsa04060 | KEGG Pathway | Cytokine-cytokine receptor interaction | -9.41 | -6.72 | 25 | 9.26 |
| hsa04650 | KEGG Pathway | Natural killer cell mediated cytotoxicity | -6.21 | -3.82 | 14 | 10.53 |
| hsa04062 | KEGG Pathway | Chemokine signaling pathway | -5.94 | -3.72 | 16 | 8.79 |
| hsa04640 | KEGG Pathway | Hematopoietic cell lineage | -5.32 | -3.27 | 11 | 11.34 |
| hsa04210 | KEGG Pathway | Apoptosis | -5.27 | -3.27 | 13 | 9.42 |
| hsa04660 | KEGG Pathway | T cell receptor signaling pathway | -5.07 | -3.27 | 11 | 10.68 |
| hsa04514 | KEGG Pathway | Cell adhesion molecules (CAMs) | -5.06 | -3.27 | 13 | 9.03 |
| hsa04380 | KEGG Pathway | Osteoclast differentiation | -4.82 | -3.12 | 12 | 9.23 |
| hsa05323 | KEGG Pathway | Rheumatoid arthritis | -4.02 | -2.43 | 9 | 10.00 |
| hsa05166 | KEGG Pathway | HTLV-I infection | -3.56 | -2.10 | 15 | 5.86 |
| hsa04666 | KEGG Pathway | Fc gamma R-mediated phagocytosis | -3.25 | -1.82 | 8 | 8.79 |
| hsa04612 | KEGG Pathway | Antigen processing and presentation | -3.00 | -1.61 | 7 | 9.09 |
| hsa04623 | KEGG Pathway | Cytosolic DNA-sensing pathway | -2.74 | -1.43 | 6 | 9.52 |
| hsa05321 | KEGG Pathway | Inflammatory bowel disease (IBD) | -2.67 | -1.38 | 6 | 9.23 |
| hsa05202 | KEGG Pathway | Transcriptional misregulation in cancer | -2.39 | -1.13 | 10 | 5.56 |
| hsa05200 | KEGG Pathway | Pathways in cancer | -2.11 | -0.91 | 16 | 4.05 |

Abbreviations: PKP: plakophilin; KEGG: Kyoto Encyclopedia of Genes and Genomes.; OV: Ovarian serous cystadenocarcinoma.

**Table S7 Significantly enriched GO (Cellular Components) analysis of PKP3 and co-expression genes in OV (Metascape).**

| **Term** | **Category** | **Description** | **Log *P*** | **Log(*q*-value)** | **Count** | **%** |
| --- | --- | --- | --- | --- | --- | --- |
| GO:0005911 | GO Cellular Components | cell-cell junction | -11.61 | -8.32 | 34 | 8.56 |
| GO:0070161 | GO Cellular Components | anchoring junction | -7.08 | -4.51 | 33 | 5.84 |
| GO:0045177 | GO Cellular Components | apical part of cell | -7.02 | -4.51 | 27 | 6.67 |
| GO:0015629 | GO Cellular Components | actin cytoskeleton | -5.45 | -3.17 | 28 | 5.42 |
| GO:0016323 | GO Cellular Components | basolateral plasma membrane | -4.14 | -1.94 | 15 | 6.64 |
| GO:0030057 | GO Cellular Components | desmosome | -3.87 | -1.70 | 5 | 20.00 |
| GO:0097342 | GO Cellular Components | ripoptosome | -3.79 | -1.65 | 3 | 50.00 |
| GO:0043256 | GO Cellular Components | laminin complex | -2.90 | -0.86 | 3 | 27.27 |
| GO:0005903 | GO Cellular Components | brush border | -2.89 | -0.86 | 8 | 7.77 |
| GO:0042581 | GO Cellular Components | specific granule | -2.23 | -0.39 | 9 | 5.63 |
| GO:0031252 | GO Cellular Components | cell leading edge | -2.22 | -0.39 | 17 | 4.07 |
| GO:0045335 | GO Cellular Components | phagocytic vesicle | -2.21 | -0.39 | 8 | 6.02 |
| GO:0043034 | GO Cellular Components | costamere | -2.19 | -0.39 | 3 | 15.79 |
| GO:0005913 | GO Cellular Components | cell-cell adherens junction | -2.12 | -0.36 | 3 | 15.00 |
| GO:0045121 | GO Cellular Components | membrane raft | -2.10 | -0.35 | 14 | 4.28 |
| GO:0048471 | GO Cellular Components | perinuclear region of cytoplasm | -2.06 | -0.35 | 25 | 3.44 |
| GO:0005922 | GO Cellular Components | connexin complex | -2.06 | -0.35 | 3 | 14.29 |

Abbreviations: PKP: plakophilin; GO: Gene Ontology; OV: Ovarian serous cystadenocarcinoma.

**Table S8 Significantly enriched GO (Molecular Function) analysis of PKP3 and co-expression genes in OV (Metascape).**

| **Term** | **Category** | **Description** | **Log *P*** | **Log(*q*-value)** | **Count** | **%** |
| --- | --- | --- | --- | --- | --- | --- |
| GO:0050839 | GO Molecular Functions | cell adhesion molecule binding | -8.83 | -5.16 | 34 | 6.75 |
| GO:0019904 | GO Molecular Functions | protein domain specific binding | -6.14 | -2.78 | 36 | 5.06 |
| GO:0003779 | GO Molecular Functions | actin binding | -4.03 | -0.97 | 22 | 5.10 |
| GO:0004252 | GO Molecular Functions | serine-type endopeptidase activity | -3.82 | -0.86 | 12 | 7.32 |
| GO:0017137 | GO Molecular Functions | Rab GTPase binding | -3.65 | -0.77 | 12 | 7.02 |
| GO:0098641 | GO Molecular Functions | cadherin binding involved in cell-cell adhesion | -3.37 | -0.65 | 4 | 22.22 |
| GO:0008376 | GO Molecular Functions | acetylgalactosaminyltransferase activity | -3.37 | -0.65 | 6 | 12.50 |
| GO:0005548 | GO Molecular Functions | phospholipid transporter activity | -3.32 | -0.65 | 6 | 12.24 |
| GO:0005178 | GO Molecular Functions | integrin binding | -3.27 | -0.65 | 10 | 7.30 |
| GO:1901612 | GO Molecular Functions | cardiolipin binding | -2.90 | -0.35 | 3 | 27.27 |
| GO:0038024 | GO Molecular Functions | cargo receptor activity | -2.86 | -0.35 | 7 | 8.64 |
| GO:0097493 | GO Molecular Functions | structural molecule activity conferring elasticity | -2.79 | -0.32 | 3 | 25.00 |
| GO:0030165 | GO Molecular Functions | PDZ domain binding | -2.71 | -0.28 | 7 | 8.14 |
| GO:0015293 | GO Molecular Functions | symporter activity | -2.49 | -0.15 | 9 | 6.16 |
| GO:0000978 | GO Molecular Functions | RNA polymerase II proximal promoter sequence-specific DNA binding | -2.45 | -0.13 | 23 | 3.81 |
| GO:0001161 | GO Molecular Functions | intronic transcription regulatory region sequence-specific DNA binding | -2.41 | -0.12 | 3 | 18.75 |
| GO:0005243 | GO Molecular Functions | gap junction channel activity | -2.26 | -0.05 | 3 | 16.67 |
| GO:0060090 | GO Molecular Functions | molecular adaptor activity | -2.16 | 0.00 | 12 | 4.69 |
| GO:1901505 | GO Molecular Functions | carbohydrate derivative transmembrane transporter activity | -2.05 | 0.00 | 4 | 10.00 |

Abbreviations: PKP: plakophilin; GO: Gene Ontology; OV: Ovarian serous cystadenocarcinoma.

**Table S9 Significantly enriched GO (Biological Processes) analysis of PKP3 and co-expression genes in OV (Metascape).**

| **Term** | **Category** | **Description** | **Log *P*** | **Log(*q*-value)** | **Count** | **%** |
| --- | --- | --- | --- | --- | --- | --- |
| GO:0045216 | GO Biological Processes | cell-cell junction organization | -9.83 | -5.63 | 20 | 12.35 |
| GO:0030029 | GO Biological Processes | actin filament-based process | -5.99 | -2.74 | 38 | 4.84 |
| GO:0030198 | GO Biological Processes | extracellular matrix organization | -5.49 | -2.38 | 23 | 6.17 |
| GO:0070268 | GO Biological Processes | cornification | -5.44 | -2.38 | 12 | 10.62 |
| GO:0030260 | GO Biological Processes | entry into host cell | -5.40 | -2.38 | 13 | 9.70 |
| GO:0014020 | GO Biological Processes | primary neural tube formation | -5.27 | -2.30 | 11 | 11.22 |
| GO:0034341 | GO Biological Processes | response to interferon-gamma | -4.71 | -1.92 | 15 | 7.43 |
| GO:0046640 | GO Biological Processes | regulation of alpha-beta T cell proliferation | -4.47 | -1.71 | 6 | 19.35 |
| GO:1990778 | GO Biological Processes | protein localization to cell periphery | -4.36 | -1.65 | 19 | 5.88 |
| GO:0043616 | GO Biological Processes | keratinocyte proliferation | -4.29 | -1.61 | 7 | 14.58 |
| GO:0002758 | GO Biological Processes | innate immune response-activating signal transduction | -4.26 | -1.61 | 18 | 5.98 |
| GO:0007163 | GO Biological Processes | establishment or maintenance of cell polarity | -4.19 | -1.58 | 15 | 6.70 |
| GO:0031581 | GO Biological Processes | hemidesmosome assembly | -4.12 | -1.54 | 4 | 33.33 |
| GO:0050891 | GO Biological Processes | multicellular organismal water homeostasis | -4.09 | -1.53 | 8 | 11.59 |
| GO:0007264 | GO Biological Processes | small GTPase mediated signal transduction | -4.03 | -1.50 | 26 | 4.68 |
| GO:1901264 | GO Biological Processes | carbohydrate derivative transport | -3.71 | -1.29 | 8 | 10.26 |
| GO:0009611 | GO Biological Processes | response to wounding | -3.62 | -1.21 | 29 | 4.20 |
| GO:0016266 | GO Biological Processes | O-glycan processing | -3.61 | -1.21 | 7 | 11.48 |
| GO:1900027 | GO Biological Processes | regulation of ruffle assembly | -3.48 | -1.09 | 5 | 16.67 |
| GO:0014908 | GO Biological Processes | myotube differentiation involved in skeletal muscle regeneration | -3.35 | -1.00 | 3 | 37.50 |

Abbreviations: PKP: plakophilin; GO: Gene Ontology; OV: Ovarian serous cystadenocarcinoma

**Table S10 Significantly enriched KEGG pathway analysis of PKP3 and co-expression genes in OV (Metascape).**

| **Term** | **Category** | **Description** | **Log *P*** | **Log(*q*-value)** | **Count** | **%** |
| --- | --- | --- | --- | --- | --- | --- |
| hsa04530 | KEGG Pathway | Tight junction | -5.62 | -2.92 | 15 | 8.82 |
| hsa04621 | KEGG Pathway | NOD-like receptor signaling pathway | -4.29 | -1.90 | 13 | 7.65 |
| hsa05160 | KEGG Pathway | Hepatitis C | -3.42 | -1.25 | 10 | 7.63 |
| hsa04622 | KEGG Pathway | RIG-I-like receptor signaling pathway | -3.24 | -1.25 | 7 | 10.00 |
| hsa04520 | KEGG Pathway | Adherens junction | -2.45 | -0.54 | 6 | 8.33 |
| hsa04390 | KEGG Pathway | Hippo signaling pathway | -2.34 | -0.54 | 9 | 5.84 |
| hsa04512 | KEGG Pathway | ECM-receptor interaction | -2.17 | -0.43 | 6 | 7.32 |

Abbreviations: PKP: plakophilin; KEGG: Kyoto Encyclopedia of Genes and Genomes.; OV: Ovarian serous cystadenocarcinoma.

**Table S11 Expression of PKP2 in different ovarian tissues including 53 high grade ovarian cancer**

| Groups | Cases | Low | | High | | Positive Rate(%) | High expression Rate (%) |
| --- | --- | --- | --- | --- | --- | --- | --- |
|  |  | **-** | **+** | **++** | **+++** |  |  |
| Malignant | 53 | 4 | 12 | 24 | 13 | 92.4% ^a,b,c^ | 69.8% ^d,e,f^ |
| Borderline | 23 | 13 | 2 | 4 | 4 | 43.5% | 34.8% ^g^ |
| Benign | 15 | 11 | 1 | 1 | 2 | 26.7% | 20.0% |
| Normal | 15 | 12 | 1 | 2 | 0 | 20.0% | 13.3% |

**Note:** ^a,b,c^ indicated that the positive expression rate of PKP2 in malignant tissues was compared with that in borderline group, benign group and normal tissues, all *P*＜0.05 (*P*_a_＜0.001, *P*_b_＜0.001, *P*_c_＜0.001); ^d,e,f^ indicated that the high positive rate of PKP2 in malignant tissues is higher than that in borderline group, benign group and normal tissues, all *P*＜0.05 (*P*_d_=0.004, *P*_e_=001, *P*_e_＜0.001); ^g^ indicated that the high positive expression rate of PKP2 in borderline tissues was compared with that in normal tissues, *P*＜0.05(*P*_g_=0.030).

**Table S12 Relationships between the expression of PKP2 and clinicopathological parameters of 53 high grade ovarian cancer patients**

| Groups | Cases | Low | |  | High | | Positive rate(%) | *P-*value | High expression rate(%) | *P-*value |
| --- | --- | --- | --- | --- | --- | --- | --- | --- | --- | --- |
|  |  | (-) | (+) |  | (++) | (+++) |  |  |  |  |
| **Age at diagnosis** |  |  |  |  |  |  |  |  |  |  |
| <59 | 31 | 1 | 7 |  | 16 | 7 | 96.8% | *P*=0.376 | 74.2% | *P*=0.409 |
| ≥59 | 22 | 3 | 5 |  | 8 | 6 | 86.4% |  | 63.6% |  |
| **Pathological type** |  |  |  |  |  |  |  |  |  |  |
| Serous | 42 | 4 | 9 |  | 21 | 8 | 90.5% | *P*=0.378 | 69.0% | *P*=0.936 |
| Endometrioid | 3 | 0 | 1 |  | 1 | 1 | 100.0% |  | 66.7% |  |
| Clear cell carcinoma | 8 | 0 | 2 |  | 2 | 4 | 100.0% |  | 75.0% |  |
| **FIGO stage** |  |  |  |  |  |  |  |  |  |  |
| I-II | 20 | 2 | 6 |  | 6 | 6 | 90.0% | *P*=1 | 60.0% | *P*=0.226 |
| III-IV | 33 | 2 | 6 |  | 18 | 7 | 93.9% |  | 75.8% |  |
| **Lymphatic metastasis** |  |  |  |  |  |  |  |  |  |  |
| No | 30 | 3 | 10 |  | 11 | 6 | 90.0% | *P*=1 | 56.7% | *P*=0.265 |
| Yes | 11 | 1 | 1 |  | 5 | 4 | 90.9% |  | 81.8% |  |
| Unknown | 12 | 0 | 1 |  | 8 | 3 | 100.0% |  | 91.7% |  |
